# Isolation, Structure Elucidation and Biological Evaluation of Lagunamide D: A New Cytotoxic Macrocyclic Depsipeptide from Marine Cyanobacteria

**DOI:** 10.3390/md17020083

**Published:** 2019-02-01

**Authors:** Danmeng Luo, Masteria Y. Putra, Tao Ye, Valerie J. Paul, Hendrik Luesch

**Affiliations:** 1 Department of Medicinal Chemistry and Center for Natural Products, Drug Discovery and Development (CNPD3), University of Florida, Gainesville, FL 32610, USA; dmluo@ufl.edu (D.L.); MPutra@cop.ufl.edu (M.Y.P.); 2 Research Center for Oceanography, Indonesian Institute of Sciences, Jl. Pasir Putih I, Ancol Timur, Jakarta 14430, Indonesia; 3 State Key Laboratory of Chemical Oncogenomics, Key Laboratory of Chemical Genomics, Shenzhen Graduate School of Peking University, Shenzhen 518055, China; yet@pkusz.edu.cn; 4 QianYan Pharmatech Limited, Shenzhen 518172, China; 5 Smithsonian Marine Station, 701 Seaway Drive, Fort Pierce, FL 34949, USA; paul@si.edu

**Keywords:** marine cyanobacteria, cyclic depsipeptides, acyl migration, anticancer, apoptosis

## Abstract

Lagunamide D, a new cytotoxic macrocyclic depsipeptide, was discovered from a collection of marine cyanobacteria from Loggerhead Key in the Dry Tortugas, Florida. An intramolecular ester exchange was observed, where the 26-membered macrocycle could contract to a 24-membered compound via acyl migration at the 1,3-diol unit, and the transformation product was named lagunamide D’. The planar structures of both compounds were elucidated using a combination of nuclear magnetic resonance (NMR) spectroscopy and high-resolution mass spectroscopy (HRMS). The absolute configurations were determined on the basis of enantioselective analysis, modified Mosher’s analysis, Kishi NMR database, and direct comparison with lagunamide A, a structure closely resembling lagunamide D. Lagunamides A and D displayed low-nanomolar antiproliferative activity against A549 human lung adenocarcinoma cells, while the structural transformation from the 26-membered lagunamide D macrocycle to the 24-membered ring structure for lagunamide D’ led to a 9.6-fold decrease in activity. Lagunamide D also displayed potent activity in triggering apoptosis in a dose- and time-dependent manner. Further investigation on the mechanism of action of the lagunamide scaffold is needed to fully explore its therapeutic potential as an anticancer agent.

## 1. Introduction

Marine cyanobacteria are recognized as a rich source of structurally intriguing chemical entities, which can serve as therapeutic leads to address unmet medical needs, including the treatment of cancer [1,2]. Brentuximab vedotin (Adcetris^®^), which gained the United States Food and Drug Administration (FDA) approval in 2011 for the treatment of Hodgkin lymphoma and systemic anaplastic large cell lymphoma, is the latest marketed anticancer drug derived from the ocean [3,4]. The payload of this antibody–drug conjugate was developed based on the pharmacophore of dolastatin 10, which is a marine cyanobacterial secondary metabolite displaying potency in the sub-nanomolar to picomolar range [1,5,6]. In addition to the molecules serving as templates for drug design, some compounds are at least invaluable tools enabling scientists to reveal new biology, probe new therapeutic mechanisms of action, and inspire the development of bioweapons to fight against cancer [1,7,8,9,10].

**Figure 1:**
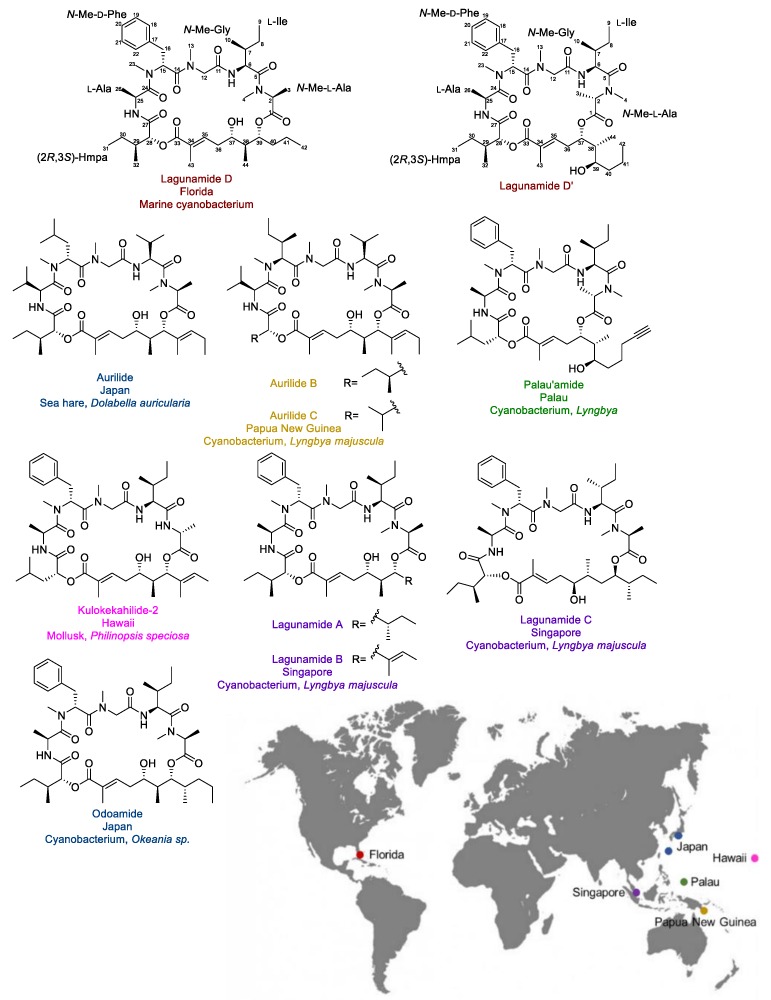
The structures, the original source organisms, the collection sites of lagunamide D and D’, and their analogues.

Lagunamide D (Figure 1) is a novel cytotoxic macrocyclic depsipeptide that was discovered from a collection of marine cyanobacteria (a mixture mainly consisting of *Dichothrix* sp. and *Lyngbya* sp. in a ratio of 1:1 with minor amount of *Rivularia* sp. present) from Loggerhead Key in the Dry Tortugas in Florida. The structure was elucidated by detailed analysis of 1D/2D NMR spectra and HRMS data. Its structure is closely related to a series of marine-originated compounds from cyanobacteria and macroorganisms known to contain or feed on cyanobacteria, including aurilides [11,12], lagunamides [13,14], kulokekahilide-2 [15], odoamide [16], and palau’amide [17] (Figure 1). As the structures of lagunamides shared the exact same peptide fragment with our newly discovered molecule, the isolated 26-membered compound was named lagunamide D. Notably, it was the first time this type of compound was identified from the Atlantic Ocean, while all the other analogues were isolated from marine organisms collected from the Pacific Ocean (the collection sites and the corresponding producers are indicated in Figure 1).

Aurilide functions in mammalian cells presumably by directly targeting prohibitin 1 (PHB1), a mitochondria inner membrane protein [18]. As the first small molecule that could interact with prohibitin, aurilide has been considered an invaluable chemical tool to reveal the biology related to prohibitin. Although structures with similar chemical skeletons are highly likely to share the same protein target, trivial structural differences can lead to distinct alterations in their target engagement and cellular functions. Therefore, the biological characterization of lagunamides is important to add more value to this family of compounds.

## 2. Results and Discussion

### 2.1. Isolation and Structure Elucidation

The freeze-dried cyanobacteria sample was extracted twice with EtOAc–MeOH (1:1) to afford the non-polar extract, which was subsequently partitioned between EtOAc and H_2_O to yield two crude fractions. The EtOAc soluble fraction was prioritized due to its greater cytotoxic activity and the crude material was applied to silica gel column chromatography for fractionation. The fraction eluting with 25% MeOH in EtOAc displayed the most potent activity and was subjected to C18 solid phase extraction (SPE) cartridge fractionation and reversed-phase high-performance liquid chromatography (HPLC) purification, yielding two semi-pure fractions that not only displayed similar NMR spectra, but also shared compounds of the same molecular weight.

**Figure 2:**
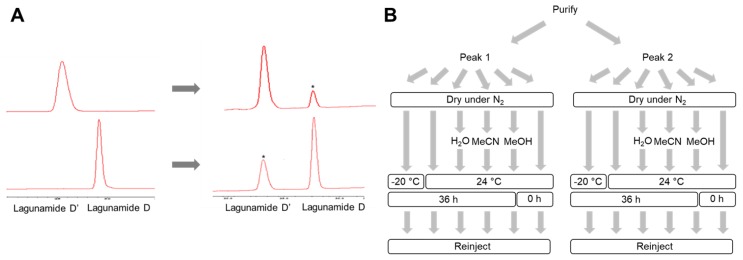
The interconversion between lagunamide D and D’. (**A**) HPLC traces indicating the interconversion between the two compounds. The converted compounds are marked by asterisks. (**B**) Work flow of the stability assessment assay.

Interestingly, during the second round of HPLC purification, an interconversion was observed between these two molecules (Figure 2A). In order to identify the cause, we investigated the impact of several factors, such as three conventionally used HPLC solvents, the time of the compound exposure to air, the temperature, and the physical states of the molecule (Figure 2B). According to our preliminary data, structural conversion was enhanced in MeOH compared with the other two HPLC solvents. We additionally found the compounds were relatively stable when stored as a solid. With this knowledge, in order to minimize the risk of structural transformation, the use of MeOH was strictly avoided in all our following studies. Although structural transformation was still detectable in MeCN, the interconversion was minimized when the exposure time in solvent was minimized. Consequently, HPLC purification was performed by loading the maximum amount of sample (around 1.5 mg) per run to purify both compounds, and each fraction was dried down immediately after each HPLC run. Acquisition of NMR spectra was performed immediately after HPLC purification, with the aim to minimize the risk of structural transformation.

The NMR data sets were acquired in (CD_3_)_2_SO using a 600 MHz spectrometer with a 5-mm probe for both molecules. Additional ^1^H NMR spectra were acquired after overnight NMR experiments to assess the stability of compounds in the (CD_3_)_2_SO solvent. No structural change was observed after 18 h exposure in (CD_3_)_2_SO at 27 °C. The ^1^H NMR spectra of both compounds displayed characteristic peptide resonances for several α-protons, secondary amide NH protons, and tertiary amide *N*-CH_3_ groups. Extensive 2D NMR analysis (Table 1 and Table 2) using HSQC, COSY, TOCSY, and HMBC established the presence of five amino acids (*N*-Me-Ala, Ile, *N*-Me-Gly, *N*-Me-Phe, and Ala) and one α-hydroxy acid 2-hydroxy-3-methylpentanoic acid (Hmpa) in both molecules. The last spin system of each compound was elucidated as a 12-carbon polyketide fragment (C-33 to C-44) with an α,β-unsaturated carbonyl system, one methine bearing a hydroxy group, and one oxygenated methine engaged in an ester linkage.

The linear sequence of *N*-Me-Ala–Ile–*N*-Me-Gly–*N*-Me-Phe–Ala–Hmpa was established based on HMBC correlations between α-protons and carbonyl groups (Table 1, lagunamide D: H-2/C-1, H-4 and H-6/C-5, H-6, NH(1), H-12a and H-12b/C-11, H-12b, H_3_-13, H-15, and H-16a/C-14, H_3_-23, H-25, H_3_-26/C-24, NH(2)/C-27; Table 2, lagunamide D’: H-2 and H-3/C-1, H-4 and H-6/C-5, NH(1), H-12a and H-12b/C-11, H-12b, H_3_-13 and H-15/C-14, H-15, H_3_-23, H-25, H_3_-26/C-24, H-25, NH(2) and H-28/C-27). In addition to the HMBC experiment optimized for*^n^J* = 7 Hz, a second HMBC experiment optimized for *^n^J* = 3 Hz was also acquired for lagunamide D’, enabling us to incorporate the dihydroxy acid polyketide fragment, furnish the two ester bonds (H-28/C-33 and H-37/C-1), and finalize the cyclic structure. All the *N*-methyl amide bonds were determined to be in *trans* conformation based on NOESY experiment. The established structures fulfilled the molecular formula of C_44_H_69_N_5_O_10_ deduced from HRMS and unsaturation number requirements (UN = 13), and we name the two compounds as lagunamide D (*m*/*z* 850.4940 for [M + Na]^+^) and lagunamide D’, (*m*/*z* 850.4946 for [M + Na]^+^).

The most distinct difference between the gross structures of the two compounds was the position of the ester bond formed between the hydroxy group of the polyketide fragment and the carbonyl group of the *N*-Me-Ala. The short distance between the two hydroxy groups (1,3-diol unit) explained the observed structural conversion during isolation. The acyl migration led to the contraction of the ring size (26-membered vs. 24-membered macrocycle). Because of the abundance of each compound (after first round of HPLC purification) and that the majority (seven out of nine) analogues were identified as 26-membered macrocycles (Figure 1). It was believed that the 26-membered macrocyclic compound, lagunamide D, should be the naturally occurring natural product. This is supported by the attenuation of cytotoxic activity during the subsequent purification process attributed to increasing abundance of the less active 24-membered macrocycle (see below). The 24-membered macrocyclic molecule was suspected to be the converted natural product and therefore termed lagunamide D’.

Enantioselective HPLC analysis coupled with UV detection and mass spectrometry of the acid hydrolysates of lagunamides D and D’ allowed us to assign the absolute configuration of all the amino acids. Except for *N*-Me-Phe, all the amino acids were in l-form. As for the α-hydroxy acid component, the four diastereomers of 2-hydroxy-3-methylpentanoic acid (Hmpa) were synthesized using the corresponding Ile isomers as starting material. Our analysis indicated that (2*R*,3*S*)-Hmpa was the diastereomer present in the macrocycle.

**Figure 3:**
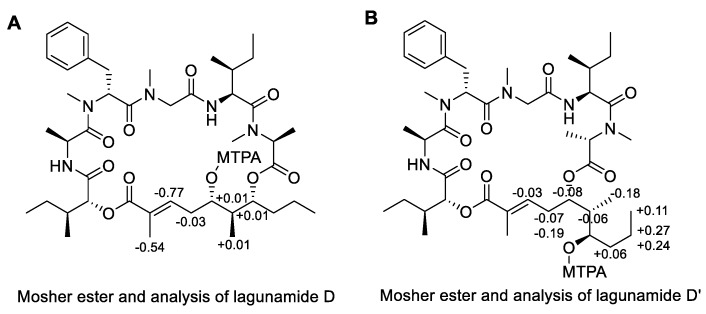
Modified Mosher’s analysis of lagunamide D (**A**) and D’ (**B**), the values of Δδ (δ_S_ − δ_R_) are shown.

**Figure 4:**
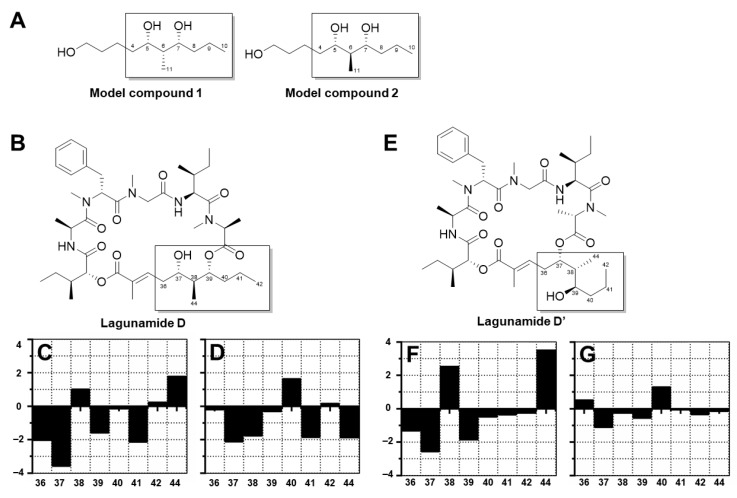
Characterization of the configuration of C-38 for lagunamide D and D’. By comparing the adjusted carbon chemical shifts of lagunamide D and D’ with the experimental carbon chemical shifts of model compounds 1 and 2, C-38 was determined to have *S* configuration, as the ^13^C NMR characteristics of the fragment of interests (the framed portion) of both lagunamide D and D’ matched better with those of model compound 2, which possessed an *S* configuration at the corresponding position. (**A**) The structure of model compounds 1 and 2. (**B**) The structure of lagunamide D. (**C**) The difference between adjusted carbon chemical shifts of lagunamide D and the experimental carbon chemical shifts of model compound 1 in (CD_3_)_2_SO. (**D**) The difference between adjusted carbon chemical shifts of lagunamide D and the experimental carbon chemical shifts of model compound 2 in (CD_3_)_2_SO. (**E**) The structure of lagunamide D’. (**F**) The difference between adjusted carbon chemical shifts of lagunamide D’ and the experimental carbon chemical shifts of model compound 1 in (CD_3_)_2_SO. (**G**) The difference between adjusted carbon chemical shifts of lagunamide D’ and the experimental carbon chemical shifts of model compound 2 in (CD_3_)_2_SO.

As for the polyketide fragment, the absolute configuration at C-37 and C-39 was determined using the modified Mosher’s method [19] and the derivatives were successfully synthesized at the 100 µg-scale. For the Mosher esters of lagunamide D and D’, the derived Δδ values (Figure 3) indicated an *S* configuration at C-37 and an *R* configuration at C-39. The stereochemistry of C-38 was deduced using the Kishi NMR database [20,21], of which two candidate model compounds (Figure 4), possessing similar structural architecture around the C-38 carbon with unknown stereochemistry in the natural product while possessing *R* or *S* configuration at the corresponding carbon in the model compounds, were employed to compare with lagunamide D and D’ (Tables S1 and S2). As depicted in Figure 4, model compound 2, the diastereomer with *S* configuration at C-38, gave a better match with the ^13^C NMR chemical shifts of both compounds. Especially for lagunamide D’, as the targeted fragment is not embedded in the macrocycle and therefore the structural flexibility is more similar to the linear type model compounds, the prediction is proposed to be more reliable. Because C-37–C-39 of lagunamide D is located in the ring system (with higher rigidity), the effect of the chemical environment would be more pronounced as a result. In addition, direct comparison of the ^1^H spectrum of lagunamide D with lagunamide A (synthetic) was performed. The same peak shape and splitting pattern of the corresponding hydrogens in the ^1^H NMR of the polyketide fragment (Figures S1–S4) further indicated that these two molecules shared the same configuration. The characterized configuration also agreed with all the other reported lagunamide analogues, which could be viewed as additional evidence to support the assignment of 37*S*,38*S*,39*R*. It is also worth noting that the complete stereochemical assignment of lagunamide D and D’ was achieved at microgram-scale (by consuming only 300 µg of compound in total for each molecule), which saved the precious natural material for biological studies.

Notably, the acyl migration was previously observed for kulokekahilide-2 (Figure 1). It was concluded that the structural transformation only occurred in the low polarity solvent (CD_2_Cl_2_, CDCl_3_), rather than in the polar solvents (CD_3_OD, and (CD_3_)_2_SO). In addition, the d-configuration at the C-2 position was proposed to be essential for the ester exchange, as the structural transformation was not detected with the isomer bearing an l-Ala at the corresponding position [22]. However, in the case of lagunamide D, which has an l-configuration at the C-2 position, the ester migration was still observed. More recently, this intramolecular transesterification was also reported for one other analogue, odoamide (Figure 1), which possesses the same *N*-Me-l-Ala moiety as lagunamide D [23]. This evidence suggests that in addition to the configuration at the C-2 position, the presence of an *N*-Me-Ala instead of an Ala might potentially lead to a change of the conformation of the macrocycle, which could also be a factor in affecting the structural conversion.

### 2.2. In Vitro Biological Evaluation

**Figure 5:**
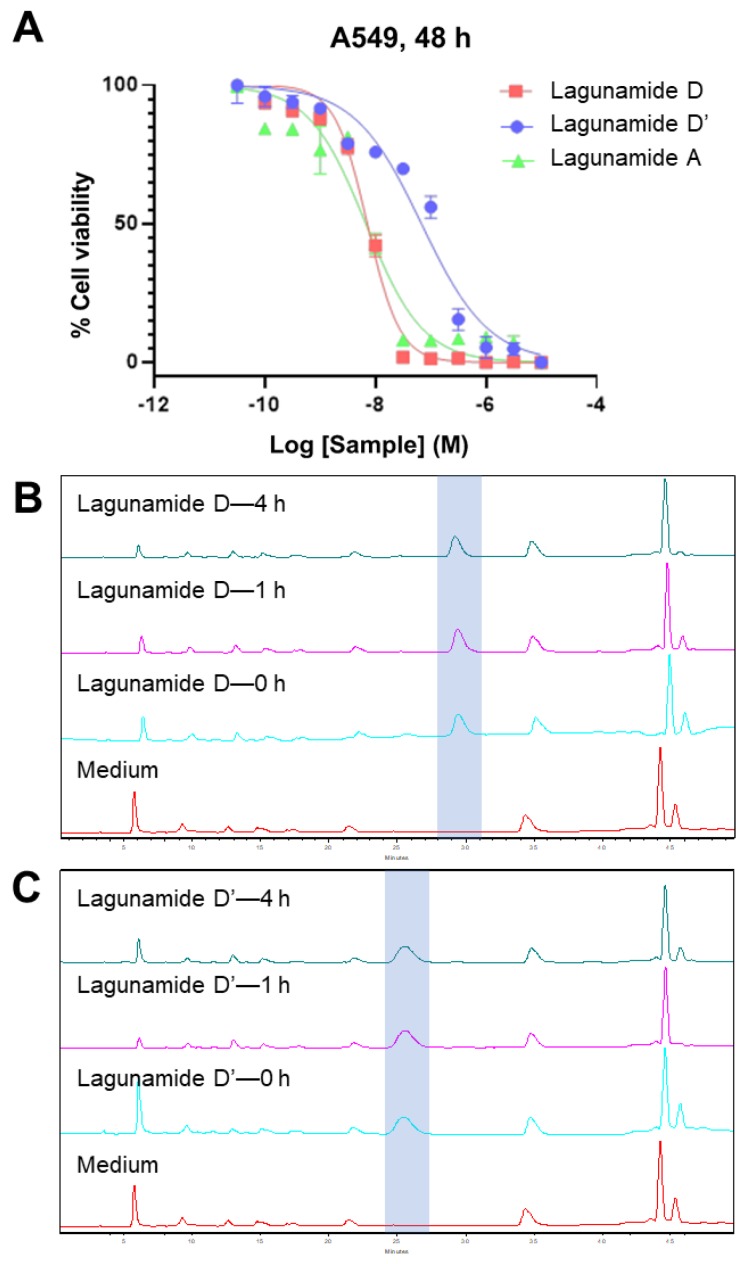
The antiproliferative effect of lagunamide D and D’ against A549 cells in comparison with lagunamide A, and the stability of lagunamide D and D’ in DMEM. (**A**) The IC_50_ of lagunamide D was 7.1 ± 1.7 nM, which was comparable with lagunamide A 6.7 ± 2.2 nM. The IC_50_ of lagunamide D’ was 68.2 ± 2.6 nM, which displayed a 9.6-fold decrease in potency. Cells were treated with varying doses of lagunamides for 48 h, and cell viability was measured using MTT assay. Data are presented as mean ± SD (*n* = 3), relative to 0.5% DMSO treatment. (**B**,**C**) The HPLC traces (detected at 200 nm) of lagunamide D (**B**) and lagunamide D’ (**C**) after incubating in cell culture medium (DMEM supplemented with 10% FBS and 1% penicillin-streptomycin) under a humidified environment with 5% CO_2_ at 37 °C after different periods of time. Lagunamide D and D’ remained intact after 4 h of incubation in cell culture medium.

The antiproliferative activity of lagunamide D was evaluated in A549 human lung adenocarcinoma cells using MTT assays, and the determined IC_50_ was at single-digit nanomolar range (7.1 ± 1.7 nM, Figure 5A), which was comparable with lagunamide A (6.7 ± 2.2 nM, Figure 5A). As lagunamide D’ possessed distinct structural features compared with the parent compound lagunamide D, a structure-activity relationship (SAR) study was subsequently performed. A 9.6-fold decrease in potency was observed between the 26-membered lagunamide D and the 24-membered lagunamide D’ (Figure 5A), which implied the importance of the ring size (both lagunamide D and D’ remained intact after 4 h in DMEM supplemented with 10% FBS and 1% penicillin-streptomycin, Figure 5B,C). However, according to previous reports, the potencies of kulokekahilide-2 [22] and odoamide [23] were similar to those of their corresponding 24-membered macrocyclic acyl migration products. This discrepancy gave us a hint that different cellular effects might exist among analogues with minute structural differences.

**Figure 6:**
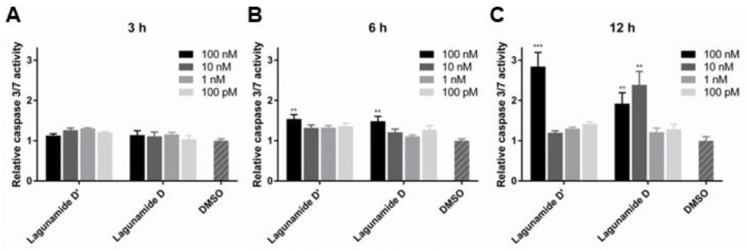
The effect of lagunamides D and D’ in triggering apoptosis in A549 cells. The activity of caspase 3/7 was measured after treating with lagunamides D and D’ for 3 h (**A**), 6 h (**B**) and 12 h (**C**), respectively. The activity of caspase 3/7 was induced after treating with lagunamides D and D’ for 6 and 12 h. Data are presented as mean ± SD (*n* = 3), ** *p* < 0.01, *** *p* < 0.001, relative to 0.5% DMSO treatment.

Similar to other analogues in this compound family (data was reported for aurilide [18], aurilide B [24], and lagunamide A [25,26]), lagunamide D and D’ were also demonstrated to trigger the apoptosis pathway (Figure 6). Both compounds possess the ability to rapidly induce apoptosis, evidenced by the activation of caspase 3/7 after only 6 h of treatment. More significant induction of apoptosis could be detected at 12 h for both lagunamide D and D’, and their difference in potency could also be clearly observed.

## 3. Materials and Methods

### 3.1. General

Flash column chromatography was performed with Fisher 170−400 mesh silica gel with the indicated solvent system. The HPLC-based compound purification was performed on a Shimadzu LC-20AB prominence liquid chromatography system with peak detection by a Shimadzu SPD-20A prominence UV/VIS detector (UV detection at 200 and 220 nm). The ^1^H and 2D nuclear magnetic resonance (NMR) spectra were recorded in (CD_3_)_2_SO on a Bruker Avance II 600 MHz spectrometer equipped with a 5-mm TXI cryogenic probe using residual solvent signals (*δ*_H_ 2.50; *δ*_C_ 39.52) as internal standards. The HSQC and HMBC experiments were optimized for ^1^*J*_CH_ = 145 and ^n^*J*_CH_ = 7 or 3 Hz, respectively. The TOCSY experiments were done using a mixing time of 100 ms. Chemical shifts (*δ*) were reported in parts per million (ppm). Multiplicities were given as the following abbreviations: s (singlet), d (doublet), q (quartet), m (multiplet), dd (doublet of doublets), ddd (doublet of doublet of doublets), qd (quartet of doublet), br s (broad singlet), and br d (broad doublet). Coupling constants (*J*) are reported in Hertz (Hz). High-resolution mass spectroscopy (HRMS) was performed using an Agilent-LC-TOF mass spectrometer with an APCI/ESI multimode ion source detector.

### 3.2. Biological Material

The dark-brown encrusting cyanobacterial tufts (DRTO 85) were collected from the shallow reef at Loggerhead Key, FL, on 9 May 2015 and were kept frozen after collection. The sample was examined microscopically and identified as a mixture of *Dichothrix* sp. (dark green tufts, 52% of the sample wet weight, thick sheath was clear to amber in color with tapering trichomes and false branches with basal heterocysts; cells nearly cuboidal though slightly wider than they were tall; trichome width = 30–55 μm, cell width = 12–30 μm, cell length = 10–25 μm), *Lyngbya* sp. (red filaments, 43% of the wet weight, sheath was nearly indistinct, cells were disc shaped and densely stacked, very consistent in size throughout the filament; trichome/cell width: 12.5 μm, cell length: 2.5 μm, with red algae *Ceramium* sp. serving as scaffold), and *Rivularia* sp. (green blobs, 5% of the wet weight, trichome width: 7.5–10 μm, cell width at base: 7.5 μm, cell length at base: 7.5–10 μm). A voucher specimen was maintained at the Smithsonian Marine Station, Fort Pierce, FL, USA. The frozen cyanobacteria sample was lyophilized and weighed prior to extraction.

### 3.3. Extraction and Isolation

The freeze-dried material (71.30 g) was extracted twice with EtOAc–MeOH (1:1) to yield the nonpolar extract (9.22 g). This was partitioned between EtOAc and H_2_O, and the EtOAc soluble fraction was concentrated to dryness (656.73 mg) and chromatographed on a column of Si gel eluted first with hexanes, followed by increasing concentrations of EtOAc; after 100% EtOAc, increasing gradients of MeOH were used. The 25% MeOH/EtOAc fraction (233.96 mg) was subjected to a SNAP C18 12g cartridge (Biotage, Uppsala, Sweden) eluted first with 25% MeOH/H_2_O, followed by increasing concentrations of MeOH; after 100% MeOH, EtOAc, and CH_2_Cl_2_ were used to flush the column. The 100% MeOH C18 fraction was further purified by semipreparative reversed-phase HPLC (column, Phenomenex Synergi 4 μ Hydro-RP 80 Å, 250 × 10 mm, 4 μm; flow rate, 2.0 mL/min; UV detection at 200 nm and 220 nm) using a linear gradient of MeOH–H_2_O (80–90% MeOH in 30 min, 100% MeOH for 15 min, and then 100%–80% MeOH in 10 min) to yield semi-pure lagunamide D and D’. The final purification was achieved using the similar chromatographic condition with a different solvent system and a different linear gradient of MeCN–H_2_O (60%–84% MeCN in 36 min, 100% MeCN for 10 min, and then 100%–60% MeCN in 10 min) to yield lagunamide D (*t*_R_ 29.0 min, 2.27 mg) and D’ (*t*_R_ 25.5 min, 1.58 mg) as colorless, amorphous solids. For lagunamide D, [α]^20^_D_–34.7 (*c* 0.05, MeOH); ^1^H NMR, COSY, HSQC, HMBC data, see Table 1; HRMS *m/z* 850.4940 [M + Na]^+^ (calcd for C_44_H_69_N_5_O_10_Na, 850.4942). For lagunamide D’, [α]^20^_D_–35.3 (*c* 0.10, MeOH); ^1^H NMR, COSY, HSQC, HMBC data, see Table 2; HRMS *m/z* 850.4946 [M + Na]^+^ (calcd for C_44_H_69_N_5_O_10_Na, 850.4942).

### 3.4. Stability Assessment

The same amounts (20 µg) of the freshly isolated lagunamide D and D’ were dispensed into different tubes and immediately dried down under N_2_. One fraction was immediately injected into the HPLC to assess the initial composition and purity of each sample. Three fractions were incubated in MeOH, MeCN, and H_2_O, respectively, at room temperature for 36 h to assess the stability in HPLC solvent. Two fractions were kept as solid in −20 °C freezer or at room temperature for 36 h to assess the effect of different storage conditions. At the end of incubation under different conditions, samples were re-injected into HPLC to check the changes of the components. The detailed work flow is displayed in Figure 2B. The HPLC method: column (Phenomenex Synergi 4μ Hydro-RP 80 Å column, 250 × 10 mm, 4 μm); flow rate, 2.0 mL/min; UV detection at 200 nm and 220 nm; linear gradient MeCN–H_2_O (60%–100% MeCN in 30 min,100% MeCN for 10 min, and then 100%–60% MeCN in 10 min).

### 3.5. Preparation of 2-Hydroxy-3-methylpentanoic Acid (Hmpa)

To a stirring solution of l-Ile (10 mg, 0.076 mmol) in 0.2 M perchloric acid (5 mL) was added a cold (0 °C) solution of 1 M NaNO_2_ (2 mL) at 0 °C. With continued rapid stirring, the reaction mixture was allowed to reach room temperature until evolution of N_2_ subsided (about 30 min). The solution was then boiled for 3 min, cooled to room temperature, and saturated with NaCl before extraction with Et_2_O and drying under vacuum to give (2*S*,3*S*)-Hmpa (l-Hmpa). The three other stereoisomers (2*S*,3*R*)-Hmpa (l-*allo*-Hmpa), (2*R*,3*R*)-Hmpa (d-Hmpa), and (2*R*,3*S*)-Hmpa (d-*allo*-Hmpa) were synthesized in a similar manner from l-*allo*-Ile, d-Ile, and d-*allo*-Ile, respectively.

### 3.6. Acid Hydrolysis and Enantioselective Analysis

Portions of lagunamide D and D’ (100 μg) were acid-hydrolyzed (200 μL of 6 N HCl, 110 °C, 24 h), and the product mixtures were dried under N_2_, reconstituted in H_2_O, run over a prepacked 100 mg C18 cartridge (eluted with H_2_O two times and 10% MeOH one time), dried under N_2_, reconstituted with 100 µL H_2_O, and analyzed by enantioselective HPLC-UV and enantioselective HPLC-MS. The absolute configurations of the amino acids *N*-Me-Ala, *N*-Me-Phe, and Ala were determined by enantioselective HPLC-MS (column, Chirobiotic TAG (250 × 4.6 mm), Supelco; solvent, MeOH−10 mM NH_4_OAc (40:60, pH 5.33); flow rate, 0.5 mL/min; detection by ESIMS in positive ion mode (MRM scan). The acid hydrolysates of lagunamide D and D’ each showed peaks with retention times at 11.1, 38.4, and 8.1 min, corresponding to *N*-Me-l-Ala, *N*-Me-d-Phe, and l-Ala, respectively. The retention times (*t*_R_, min; MRM ion pair, parent→product) of the authentic amino acids were as follows: *N*-Me-l-Ala (11.2; 104→58), *N*-Me-d-Ala (57.0), *N*-Me-l-Phe (22.4; 180→134), *N*-Me-d-Phe (39.0), l-Ala (8.1; 90→44), d-Ala (13.4). The compound-dependent MS parameters were as follows: *N*-Me-Ala: DP 19.0, EP 7.0, CE 18.5, CXP 2.0, CEP 10.0; *N*-Me-Phe: DP 29.5, EP 4.4, CE 20.0, CXP 2.2, CEP 11.4; Ala: DP 24.0, EP 8.0, CE 16.5, CXP 6.2, CEP 11.0. The source and gas-dependent MS parameters were as follows: CUR 50, CAD medium, IS 5500, TEM 750, GS1 65, GS2 65.

In order to separate Ile isomers, the mobile phase was changed to MeOH-10 mM NH_4_OAc (90:10, pH 6.87) while the chromatographic conditions were kept the same. The acid hydrolysate of lagunamide D and D’ each showed a peak corresponding to l-Ile at 11.9 min. The retention times (*t*_R_, min; MRM ion pair, parent→product) of the authentic amino acids were as follows: l-Ile (12.2; 132→86), l-*allo*-Ile (13.2), d-Ile (60.0), d-*allo*-Ile (50.1). The compound-dependent MS parameters for Ile were as follows: DP 33.0, EP 5.4, CE 15.5, CXP 2.3, CEP 9.5.

The absolute configurations of the Hmpa were determined using enantioselective HPLC (column, CHIRALPAK MA (+) (50 × 4.6 mm); solvent, CH_3_CN−2 mM CuSO_4_ (5:95); flow rate, 1.0 mL/min; detection by UV (254 nm). The acid hydrolysates of lagunamide D and D’ each showed peaks at 15.7 min, corresponding to (2*R*,3*S*)-Hmpa. The retention times of the authentic standards were as follows: (2*S*,3*S*)-Hmpa (32.1), (2*S*,3*R*)-Hmpa (26.6), (2*R*,3*R*)-Hmpa (20.3), (2*R*,3*S*)-Hmpa (16.3). All other amino acid units eluted within 12.0 min using this chromatographic condition.

### 3.7. Modified Mosher’s Analysis

Portions of lagunamide D and D’ (100 μg) were dissolved in anhydrous DMSO (100 μL). Triethylamine (Et_3_N, 30 μL) and catalytic amount of 4-(dimethylamino)pyridine (DMAP) were added, followed by the addition of (*R*)-MTPA-Cl (3 μL) or (*S*)-MTPA-Cl (3 μL) to give the (*S*)-MTPA ester or (*R*)-MTPA ester, respectively. After stirring at room temperature for 24 h, MeOH was added to terminate the reaction. The crude reaction product was dried under N_2_ and applied onto silica SPE eluted with EtOAc–hexane (1:1). The semi-pure product was further purified by semipreparative HPLC (column, Phenomenex Synergi 4μ Hydro-RP 80 Å column, 250 × 4.68 mm, 4 μm; flow rate, 0.5 mL/min) using a linear gradient of MeCN–H_2_O (30%–100% MeCN in 30 min, 100% MeCN for 10 min, and then 100%–30% MeCN in 10 min) to yield pure (*S*)-MTPA ester and (*R*)-MTPA ester, respectively. ^1^H NMR and COSY spectra were acquired for each derivative, and the differences in chemical shift between the two MTPA esters Δδ (δ_S_ − δ_R_) were analyzed.

### 3.8. Kishi NMR Database

First, the ^13^C chemical shifts were predicted for the model compounds as well as lagunamide D using the ChemDraw program. Second, the difference of the predicted ^13^C chemical shift between a given carbon in lagunamide D and the corresponding carbon in model compounds was calculated, which reflected the potential effect of the remaining portion of the molecule on the targeted fragment. Third, the differences were subtracted from the experimental ^13^C chemical shifts of lagunamide D to create an adjusted ^13^C chemical shift profile. Finally, differences between the adjusted ^13^C chemical shifts of lagunamide D and the experimental ^13^C chemical shifts of the model compounds (two candidate diastereomers) in the same solvent (CD_3_)_2_SO were calculated to deduce the configuration of C-38 for lagunamide D. Similar procedures were performed for lagunamide D’.

### 3.9. General Cell Culture Procedure

A549 human lung adenocarcinoma cells were purchased from the American Type Culture Collection (ATCC). Cells were cultured in Dulbecco’s Modified Eagle Medium (Invitrogen Invitrogen, Carlsbad, CA, USA) supplemented with 10% fetal bovine serum (FBS, Sigma–Aldrich, St. Louis, MO, USA) and 1% penicillin-streptomycin (Invitrogen) under a humidified environment with 5% CO_2_ at 37 °C.

### 3.10. Cell Viability Assay (MTT)

A549 cells were seeded at a density of 7500 cells per well in 96-well plates. Wells with negative control (cells + medium + solvent control) and medium only (medium + solvent control) were also incorporated for determination of 100% viability and background absorbance level. After 24 h of incubation, the cells were treated with varying doses of lagunamides. The cells were incubated for corresponding hours of treatment as noted and checked under the microscope for morphology before the addition of the MTT reagent (Promega, Madison, WI, USA). Cell viability was measured according to the manufacturer’s instructions and recorded on SpectraMax M5. IC_50_ calculations were done by GraphPad Prism 6 based on triplicate experiments.

### 3.11. Stability in Cell Culture Medium

One-thousand microliters of cell culture medium (DMEM supplemented with 10% FBS and 1% penicillin-streptomycin) was first dispensed into different tubes, followed with the addition of 1 µL of lagunamide D or D’ (prepared in DMSO). The solution was then vortex-mixed for 15 s and incubated for 0 h, 1 h or 4 h. At the end of each incubation period, 400 μL of EtOAc was added to each tube, and the mixture was vigorously vortexed for 15 s, followed with centrifugation for 5 min at 1700 *g* at 4 °C. The EtOAc layer was collected and evaporated to dryness under nitrogen. Samples were reconstituted in 50 μL of MeCN and injected into the HPLC system. Blank cell culture medium was also processed using the same procedures, which served as the background. The HPLC method: column (Phenomenex Synergi 4μ Hydro-RP 80 Å column, 250 × 10 mm, 4 μm); flow rate, 2.0 mL/min; UV detection at 200 nm and 220 nm; linear gradient MeCN–H_2_O (60%–84% MeCN in 36 min, 100% MeCN for 10 min, and then 100%–60% MeCN in 10 min).

### 3.12. Caspase Activity Measurement

The A549 cells were seeded at a density of 7500 cells per well in solid, white, flat-bottom 96-well plates. Wells with negative control (cells + medium + solvent control) and medium only (medium + solvent control) were also incorporated for determination of baseline activity and background luminescence level. After 24 h of incubation, the cells were treated with varying doses of testing samples. At the end of the 3, 6, and 12 h incubation periods, 60 µL of medium was removed. The caspase-glo 3/7 reagent was prepared according to the manufacturer’s instruction (Promega) and 40 µL of reagent was added to each well. After incubation at room temperature for 1 h to ensure complete cell lysis, luminescence was measured using Envision (Perkin Elmer, Waltham, MA, USA). The relative caspase 3/7 activity of lagunamide D and D’ treated cells (with or without pan-caspase inhibitor) were compared to the solvent control.

## 4. Conclusions

In conclusion, lagunamide D, a macrocyclic depsipeptide with single-digit nanomolar anticancer activity, was discovered from a collection of marine cyanobacteria from Florida. In addition to the intact natural product, we also identified a ring-contracted 24-membered isomer as a result of an intramolecular ester exchange, and the SAR information indicated the essential role of the size of macrocycle. Moreover, both compounds also displayed potent activity in triggering apoptosis in a dose- and time-dependent manner. Results presented in this study warrant further investigation on the mechanism of action of the lagunamide scaffold to fully explore its therapeutic potential as an anticancer agent.

## Figures and Tables

**Table 1:** NMR data of lagunamide D in (CD_3_)_2_SO at 27 °C.

Unit	C/H No.	*δ*_C_ *^a^*	*δ*_H_ (*J* in Hz) *^b^*	COSY *^b^*	HMBC *^b d^*
*N*-Me-Ala	1	170.17			
	2	57.93	3.91, q (6.8)	3	1, 3, 4
	3	12.74	1.26, d (6.8)	2	1
	4	36.95	3.21, s		2, 5
Ile	5	170.88			
	6	51.07	4.92, dd (9.3, 7.7)	7, NH (1)	5, 7, 8, 10, 11
	7	37.20	1.69, ddd (10.5, 7.9, 3.1)	6, 8a, 8b, 10	
	8a	23.00	1.51, m	7, 8b, 9	
	8b		1.36, m	7, 8a, 9	
	9	10.13	0.84, m	8a, 8b	8
	10	14.55	0.89, d (6.9)	7	6, 7, 8
	NH (1)		7.21, m	6	11
*N*-Me-Gly	11	168.18			
	12a	50.38	3.98, d (−18.4)	12b	11, 13
	12b		3.31 *^c^*	12a	11, 13, 14
	13	35.65	2.73, s		12, 14
*N*-Me-Phe	14	170.18			
	15	52.33	5.28, dd (10.2, 5.3)	16a, 16b	14, 16, 17, 23
	16a	34.25	2.92, dd (−14.4, 10.3)	15, 16b	14, 15, 17, 18/22
	16b		2.77, dd (−15.0, 5.8)	15, 16a	15, 17, 18/22
	17	137.16			
	18/22	129.30	7.10, m		16, 18/22, 20
	19/21	127.51	7.15, m		17, 19/21
	20	125.93	7.13, m		18/22
	23	29.24	2.85, s		15, 24
Ala	24	172.45			
	25	44.51	4.31, qd (6.9, 6.9)	26, NH (2)	24, 26
	26	14.68	0.69, d (7.0)	25	24, 25
	NH (2)		8.49, d (6.5)	25	25, 26, 27
Hmpa	27	169.49			
	28	75.30	4.86, d (3.2)	29	29, 32
	29	36.21	1.81, m	28, 30a, 30b, 32	
	30a	25.65	1.38, m	29, 30b, 31	28, 29, 31, 32
	30b		1.25, m	29, 30a, 31	28, 29, 31, 32
	31	11.52	0.87, m	30a, 30b	29, 30
	32	14.00	0.83, m	29	28, 29, 30
Dihydroxy acid	33	168.34			
	34	126.83			
	35	144.45	7.11, m	36a, 36b, 43	
	36a	29.56	2.11, ddd (−14.4, 9.8, 9.8)	35, 36b, 37	34, 35, 37
	36b		1.89, m	35, 36a, 37	
	37	69.24	3.53, m	36a, 36b, 38, OH	
	OH		4.13, br d (4.4)	37	
	38	40.85	1.89, m	37, 39, 44	
	39	74.56	4.76, m	38, 40a, 40b	
	40a	33.62	1.56, m	40b, 39	
	40b		1.40, m	40a, 39	
	41	16.84	1.23, m	42	
	42	14.12	0.86, m	41	40, 41
	43	11.84	1.81, br s	35	33, 34, 35
	44	9.40	0.79, d (6.9)	38	37, 38, 39

*^a^* Deduced from HSQC and HMBC, 600 MHz. *^b^* 600 MHz. *^c^* Overlapping with residual water. *^d^* Optimized for *^n^J* = 7 Hz.

**Table 2:** NMR data of lagunamide D’ in (CD_3_)_2_SO at 27 °C.

Unit	C/H No.	*δ*_C_ *^a^*	*δ*_H_ (*J* in Hz) *^b^*	COSY *^b^*	HMBC *^b d e^*
*N*-Me-Ala	1	170.48			
	2	58.48	3.88, q (6.7)	3	1, 3, 4
	3	13.25	1.30, d (6.8)	2	1, 2
	4	37.46	3.22, s		2, 5
Ile	5	170.58			
	6	51.11	4.77, dd (10.1, 10.1)	7, NH (1)	5, 7, 8, 10, 11
	7	36.12	1.78, m	6, 8a, 8b, 10	
	8a	23.21	1.63, m	7, 8b, 9	7, 9
	8b		1.40, m	7, 8a, 9	7, 9
	9	11.35	0.87, m	8a, 8b	7, 8
	10	14.28	0.78, d (6.8)	7	6, 7, 8
	NH (1)		7.72, d (9.8)	6	6, 11
*N*-Me-Gly	11	167.99			
	12a	50.57	4.02, d (−18.7)	12b	11, 13
	12b		2.93, d (−18.7)	12a	11, 13, 14
	13	35.56	2.73, s		12, 14
*N*-Me-Phe	14	170.37			
	15	52.60	5.28, dd (10.0, 5.5)	16a, 16b	14, 16, 17, 23, 24
	16a	34.37	2.91, dd (−14.5, 9.9)	15, 16b	15, 17, 18/22
	16b		2.81, dd (−14.4, 5.4)	15, 16a	15, 17, 18/22
	17	137.44			
	18/22	129.21	7.11, m		16, 20
	19/21	127.61	7.16, m		17, 19/21
	20	125.99	7.13, m		18/22
	23	29.26	2.86, s		15, 24
Ala	24	173.11			
	25	44.56	4.34, qd (7.0, 7.0)	26, NH (2)	24, 26, 27
	26	14.98	0.72, d (7.1)	25	24, 25
	NH (2)		8.56, d (6.7)	25	25, 26, 27
Hmpa	27	169.87			
	28	75.66	4.83, d (2.9)	29	27, 29, 30, 32, 33 *^e^*
	29	36.31	1.83, m	28, 30a, 30b, 32	30, 32
	30a	26.12	1.39, m	29, 30b, 31	28, 29, 32
	30b		1.28, m	29, 30a, 31	28, 29, 32
	31	9.52	0.86, m	30a, 30b	29, 30
	32	13.63	0.82, m	29	28, 29, 30
Dihydroxy acid	33	168.17			
	34	127.51			
	35	140.68	6.75, ddd (6.8, 6.8, 1.2)	36a, 36b, 43	
	36a	27.98	2.66, m	35, 36b, 37	
	36b		2.36, ddd (−15.6, 7.8, 7.8)	35, 36a, 37	34, 35, 37
	37	73.79	4.85, ddd (5.0, 5.0, 5.0)	36a, 36b, 38	1 *^e^*, 35, 38, 44
	38	41.07	1.66, m	37, 39, 44	36, 37, 39, 40, 44
	39	71.00	3.31 *^c^*	38, 40, OH	
	OH		4.52, d (5.5)	39	38, 39, 40
	40	36.28	1.27, m	39	
	41a	18.47	1.42, m	41b, 42	39, 40, 42
	41b		1.25, m	41a, 42	39, 40, 42
	42	13.93	0.84, m	41	41
	43	12.03	1.85, br s	35	33, 34, 35
	44	11.12	0.76, d (7.0)	38	37, 38, 39

*^a^* Deduced from HSQC and HMBC, 600 MHz. *^b^* 600 MHz. *^c^* Overlapping with residual water. *^d^* Optimized for *^n^J* = 7 Hz if not indicated. *^e^* Optimized for *^n^J* = 3 Hz.

## Supplementary Materials

The following are available online at http://www.mdpi.com/1660-3397/17/2/83/s1, Figures S1–S4: Comparison of the ^1^H NMR spectrum of lagunamide D and A in CD_3_OD; Table S1: The difference between adjusted carbon chemical shifts of lagunamide D and those of model compounds in (CD_3_)_2_SO; Table S2: The difference between adjusted carbon chemical shifts of lagunamide D’ and those of model compounds in (CD_3_)_2_SO; and 1D/2D NMR spectra of lagunamide D and D’, Mosher ester of lagunamide D and D’.
