# Supplementary material for: Isolation, Structure Elucidation and Biological Evaluation of Lagunamide D: A New Cytotoxic Macrocyclic Depsipeptide from Marine Cyanobacteria
Source: Mar Drugs. 2019 Feb 1;17(2):83. doi: 10.3390/md17020083 (PMC6410412; doi:10.3390/md17020083)
Supplement: Supplementary file 1 [file marinedrugs-17-00083-s001.pdf]

Supplementary Materials:

# Isolation, Structure Elucidation and Biological Evaluation of Lagunamide D: A New Cytotoxic Macrocyclic Depsipeptide from Marine Cyanobacteria

**Danmeng Luo [1], Masteria Y. Putra [1,2], Tao Ye [3,4], Valerie J. Paul [5] and Hendrik Luesch [1,*]**

[1] Department of Medicinal Chemistry and Center for Natural Products, Drug Discovery and Development (CNPD3), University of Florida, Gainesville, Florida 32610, USA; dmluo@ufl.edu (D.L.); MPutra@cop.ufl.edu (M.Y.P.)
[2] Research Center for Oceanography, Indonesian Institute of Sciences, Jl. Pasir Putih I, Ancol Timur, Jakarta 14430, Indonesia
[3] State Key Laboratory of Chemical Oncogenomics, Key Laboratory of Chemical Genomics, Shenzhen Graduate School of Peking University, Shenzhen, 518055, China; yet@pkusz.edu.cn
[4] QianYan Pharmatech Limited, Shenzhen, 518172, China
[5] Smithsonian Marine Station, 701 Seaway Drive, Fort Pierce, FL 34949, USA; paul@si.edu
[*] Correspondence: luesch@cop.ufl.edu; Tel.: +1-352-273-7738

## Contents:

Figure S1. Comparison of the $^1$H spectrum of lagunamide D with lagunamide A (synthetic) in $CD_3OD$ (600 MHz) at 27 °C. .......... S3

Figure S2. Comparison of the expansion of the $^1$H spectrum ($\delta_H$ 7.10–7.35 ppm) of lagunamide D with lagunamide A (synthetic) in $CD_3OD$ (600 MHz) at 27 °C. .......... S4

Figure S3. Comparison of the expansion of the $^1$H spectrum ($\delta_H$ 3.40–5.70 ppm) of lagunamide D with lagunamide A (synthetic) in $CD_3OD$ (600 MHz) at 27 °C. .......... S5

Figure S4. Comparison of the expansion of the $^1$H spectrum ($\delta_H$ 0.70–2.40 ppm) of lagunamide D with lagunamide A (synthetic) in $CD_3OD$ (600 MHz) at 27 °C. .......... S6

Table S1. The difference between adjusted carbon chemical shifts of lagunamide D and those of model compounds in $(CD_3)_2SO$ .......... S7

Table S2. The difference between adjusted carbon chemical shifts of lagunamide D' and those of model compounds in $(CD_3)_2SO$ .......... S8

$^1$H NMR spectrum of lagunamide D in $(CD_3)_2SO$ (600 MHz) at 27 °C. .......... S9

COSY spectrum of lagunamide D in $(CD_3)_2SO$ (600 MHz) at 27 °C. .......... S10

TOCSY spectrum of lagunamide D in $(CD_3)_2SO$ (600 MHz) at 27 °C. .......... S11

NOESY spectrum of lagunamide D in $(CD_3)_2SO$ (600 MHz) at 27 °C. .......... S12

HSQC spectrum of lagunamide D in $(CD_3)_2SO$ (600 MHz) at 27 °C. .......... S13

HMBC spectrum (optimized for $^{n}J$ = 7 Hz) of lagunamide D in $(CD_3)_2SO$ (600 MHz) at 27 °C. S14

$^{1}H$ NMR spectrum of lagunamide D' in $(CD_3)_2SO$ (600 MHz) at 27 °C. S15

COSY spectrum of lagunamide D' in $(CD_3)_2SO$ (600 MHz) at 27 °C. S16

TOCSY spectrum of lagunamide D' in $(CD_3)_2SO$ (600 MHz) at 27 °C. S17

NOESY spectrum of lagunamide D' in $(CD_3)_2SO$ (600 MHz) at 27 °C. S18

HSQC spectrum of lagunamide D' in $(CD_3)_2SO$ (600 MHz) at 27 °C. S19

HMBC (optimized for $^{n}J$ = 7 Hz) spectrum of lagunamide D' in $(CD_3)_2SO$ (600 MHz) at 27 °C. S20

HMBC (optimized for $^{n}J$ = 3 Hz) spectrum of lagunamide D' in $(CD_3)_2SO$ (600 MHz) at 27 °C. S21

$^{1}H$ NMR spectrum of the (*S*)-Mosher ester of lagunamide D in $(CD_3)_2SO$ (600 MHz) at 27 °C. S22

COSY spectrum of the (*S*)-Mosher ester of lagunamide D in $(CD_3)_2SO$ (600 MHz) at 27 °C. S23

$^{1}H$ NMR spectrum of the (*R*)-Mosher ester of lagunamide D in $(CD_3)_2SO$ (600 MHz) at 27 °C. S24

COSY spectrum of the (*R*)-Mosher ester of lagunamide D in $(CD_3)_2SO$ (600 MHz) at 27 °C. S25

$^{1}H$ NMR spectrum of the (*S*)-Mosher ester of lagunamide D' in $(CD_3)_2SO$ (600 MHz) at 27 °C. S26

COSY spectrum of the (*S*)-Mosher ester of lagunamide D' in $(CD_3)_2SO$ (600 MHz) at 27 °C. S27

$^{1}H$ NMR spectrum of the (*R*)-Mosher ester of lagunamide D' in $(CD_3)_2SO$ (600 MHz) at 27 °C. S28

COSY spectrum of the (*R*)-Mosher ester of lagunamide D' in $(CD_3)_2SO$ (600 MHz) at 27 °C. S29

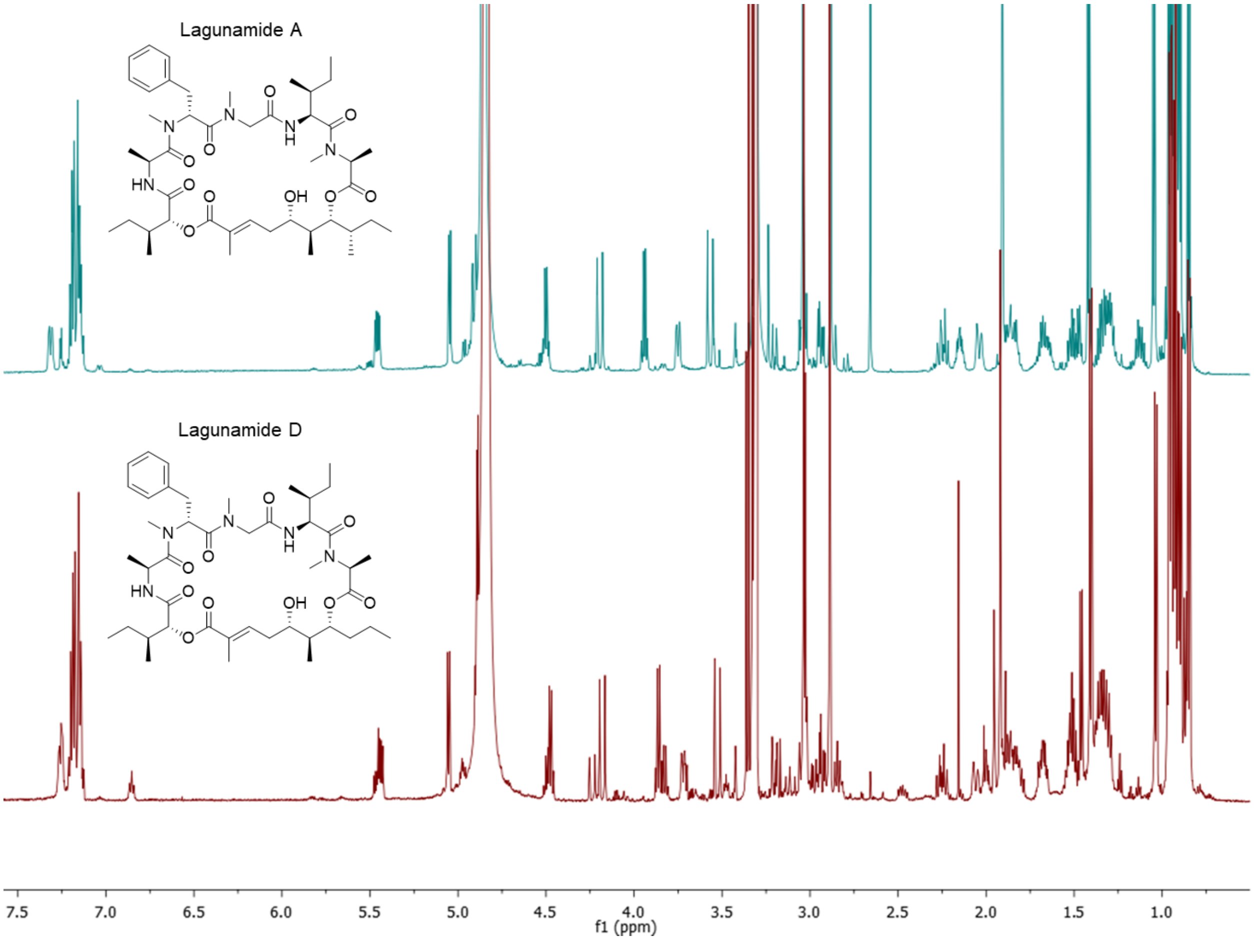


Figure S1. Comparison of the $^{1}H$ spectrum of lagunamide D with lagunamide A (synthetic) in $CD_3OD$ (600 MHz) at 27 °C.

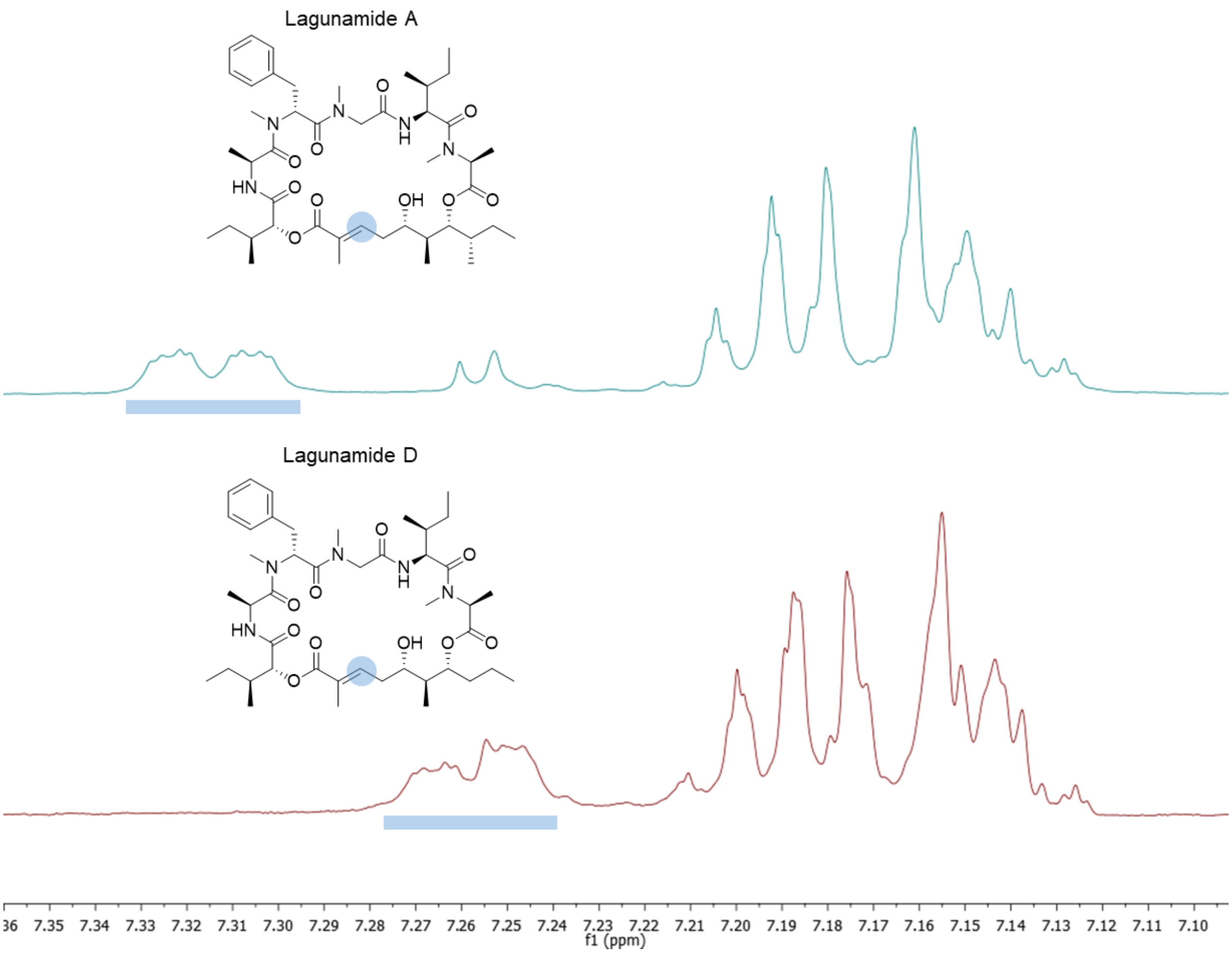


Figure S2. Comparison of the expansion of the $^{1}H$ spectrum ($\delta_H$ 7.10–7.35 ppm) of lagunamide D with lagunamide A (synthetic) in $CD_3OD$ (600 MHz) at 27 °C. The key signal and the corresponding hydrogen were highlighted in blue.

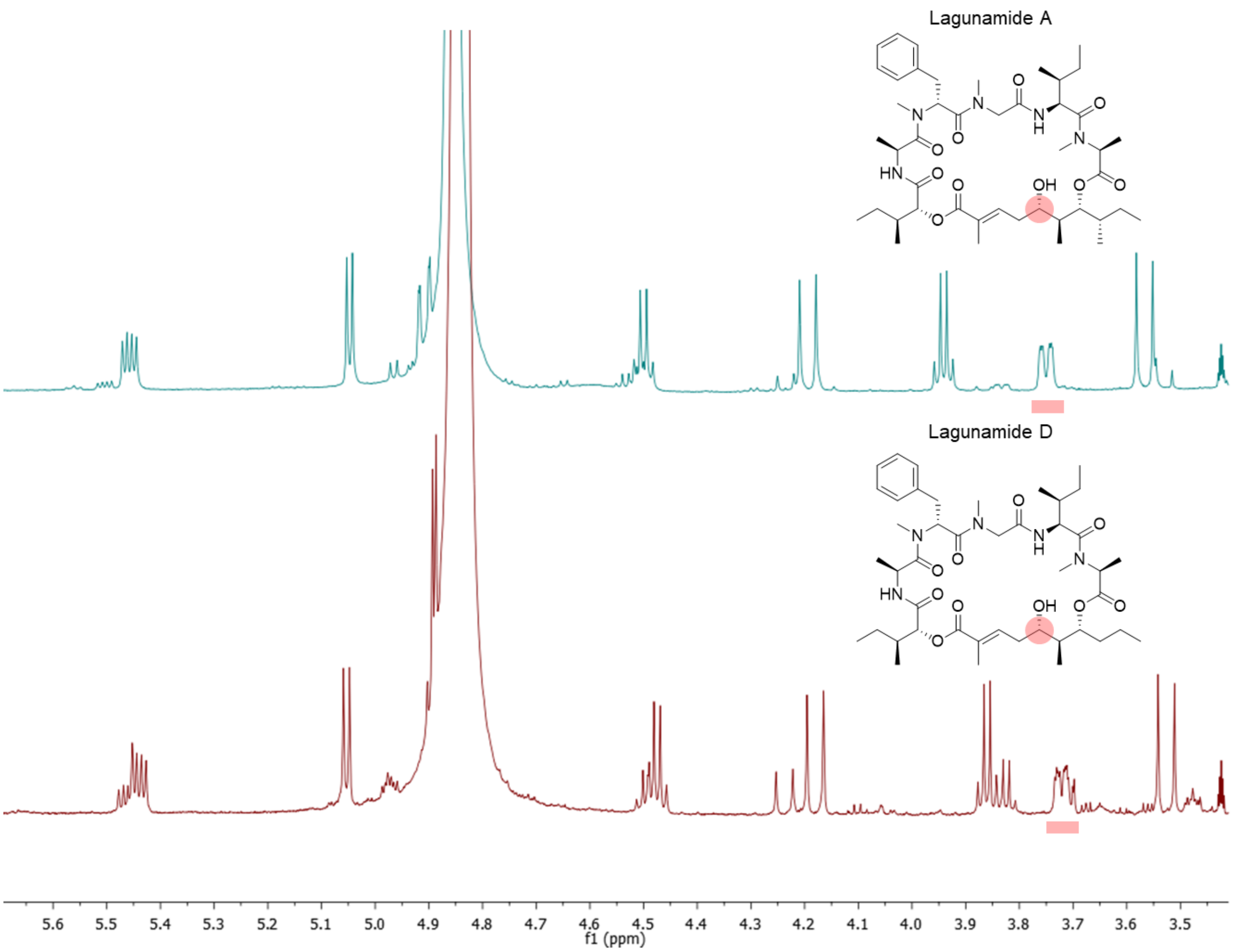


Figure S3. Comparison of the expansion of the $^1$H spectrum ($\delta_H$ 3.40–5.70 ppm) of lagunamide D with lagunamide A (synthetic) in $CD_3OD$ (600 MHz) at 27 °C. The key signal and the corresponding hydrogen were highlighted in red.

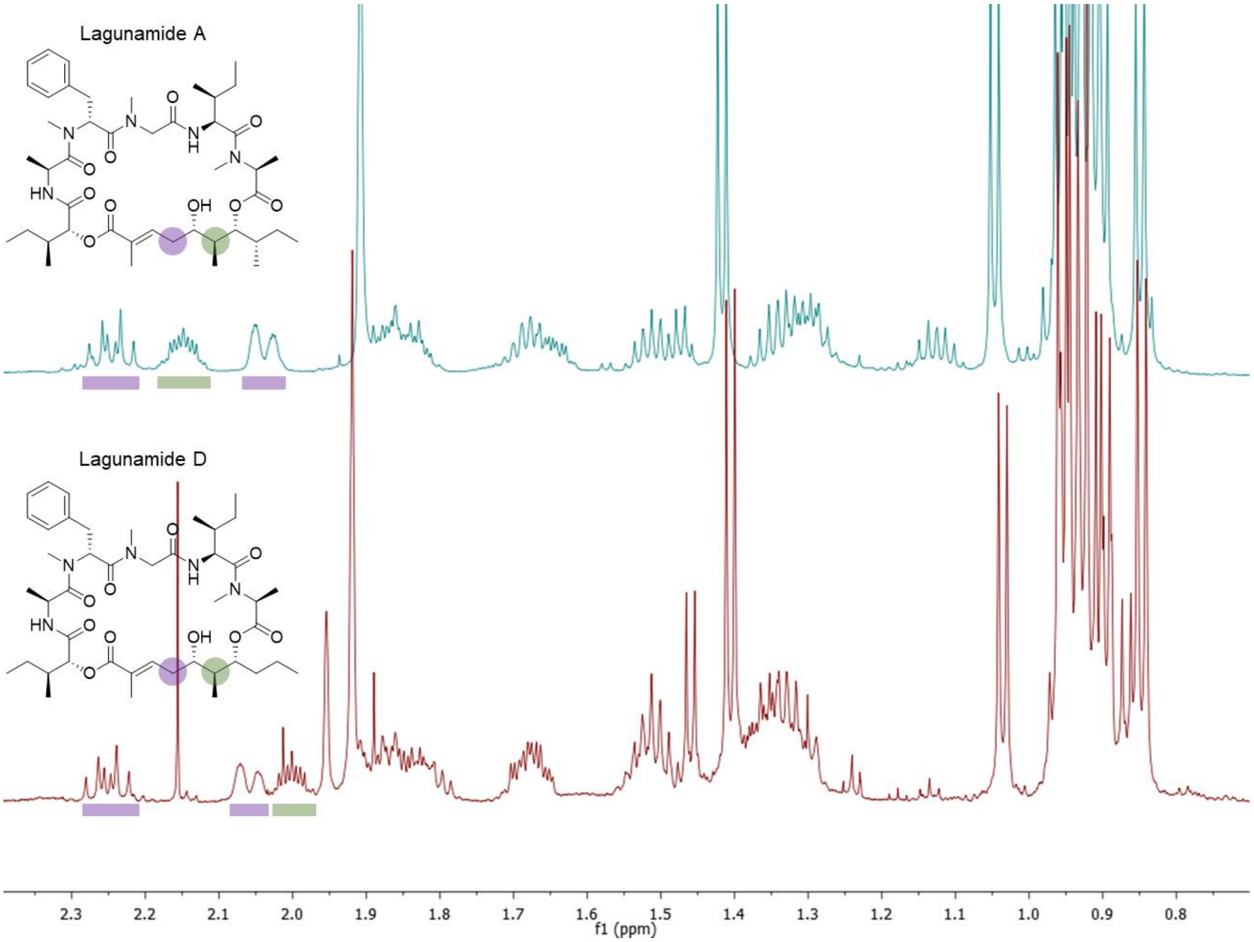


Figure S4. Comparison of the expansion of the $^{1}$H spectrum ($\delta_H$ 0.70–2.40 ppm) of lagunamide D with lagunamide A (synthetic) in $CD_3OD$ (600 MHz) at 27 °C. The key signal and the corresponding hydrogen were highlighted in green and purple.

Table S1. The difference between adjusted carbon chemical shifts of lagunamide D and those of model compounds in $(CD_3)_2SO$.

| C no. | Experimental chemical shift | | | Predicted chemical shift | | Adjusted chemical shift | Difference | |
|---|---|---|---|---|---|---|---|---|
| Lagunamide D/ model compound | Lagunamide D | Model compound 1 | Model compound 2 | Lagunamide D | Model compound | Lagunamide D | Model compound 1 | Model compound 2 |
| 36/4 | 29.56 | 34.692 | 32.856 | 31.3 | 34.4 | 32.66 | –2.032 | –0.196 |
| 37/5 | 69.28 | 72.850 | 71.400 | 70.5 | 70.5 | 69.28 | –3.57 | –2.12 |
| 38/6 | 40.86 | 41.751 | 44.519 | 44 | 45.9 | 42.76 | 1.009 | –1.759 |
| 39/7 | 74.56 | 72.544 | 71.254 | 73.8 | 70.2 | 70.96 | –1.584 | –0.294 |
| 40/8 | 33.62 | 37.067 | 35.282 | 34.5 | 37.8 | 36.92 | –0.147 | 1.638 |
| 41/9 | 16.88 | 18.819 | 18.527 | 19.3 | 19.1 | 16.68 | –2.139 | –1.847 |
| 42/10 | 14.12 | 14.186 | 14.266 | 14.1 | 14.4 | 14.42 | 0.234 | 0.154 |
| 44/11 | 9.4 | 7.316 | 10.966 | 8.9 | 8.6 | 9.1 | 1.784 | –1.866 |

Table S2. The difference between adjusted carbon chemical shifts of lagunamide D’ and those of model compounds in $(CD_3)_2SO$.

| C no. | Experimental chemical shift | | | Predicted chemical shift | | Adjusted chemical shift | Difference | |
|---|---|---|---|---|---|---|---|---|
| Lagunamide D’/ model compound | Lagunamide D’ | Model compound 1 | Model compound 2 | Lagunamide D’ | Model compound | Lagunamide D’ | Model compound 1 | Model compound 2 |
| 36/4 | 27.98 | 34.692 | 32.856 | 29 | 34.4 | 33.38 | –1.312 | 0.524 |
| 37/5 | 73.79 | 72.85 | 71.4 | 74 | 70.5 | 70.29 | –2.56 | –1.11 |
| 38/6 | 41.07 | 41.751 | 44.519 | 42.7 | 45.9 | 44.27 | 2.519 | –0.249 |
| 39/7 | 71 | 72.544 | 71.254 | 70.5 | 70.2 | 70.7 | –1.844 | –0.554 |
| 40/8 | 36.28 | 37.067 | 35.282 | 37.5 | 37.8 | 36.58 | –0.487 | 1.298 |
| 41/9 | 18.47 | 18.819 | 18.527 | 19.1 | 19.1 | 18.47 | –0.349 | –0.057 |
| 42/10 | 13.93 | 14.186 | 14.266 | 14.4 | 14.4 | 13.93 | –0.256 | –0.336 |
| 44/11 | 11.12 | 7.316 | 10.966 | 8.9 | 8.6 | 10.82 | 3.504 | –0.146 |

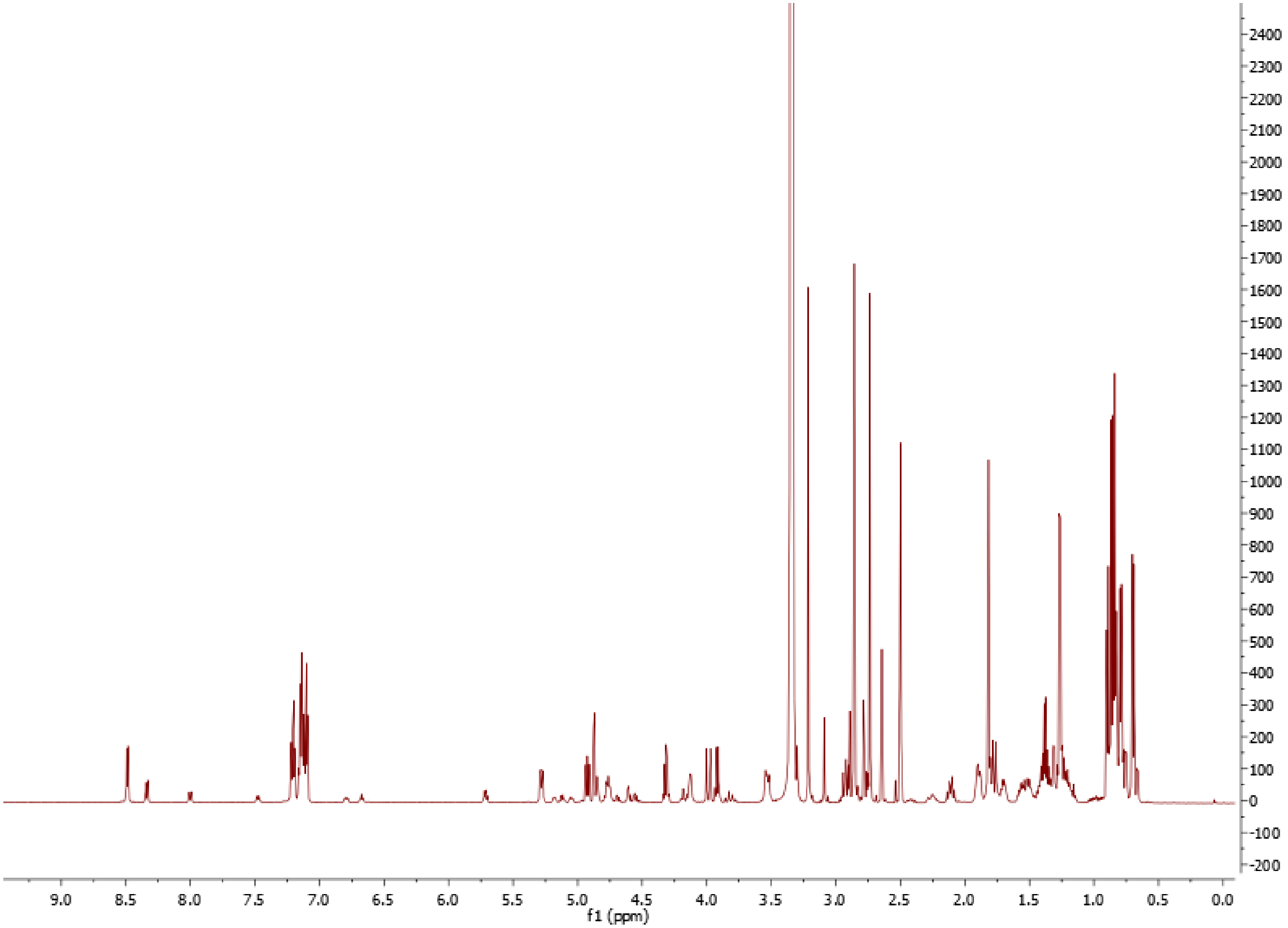


$^{1}$H NMR spectrum of lagunamide D in $(CD_3)_2SO$ (600 MHz) at 27 °C.

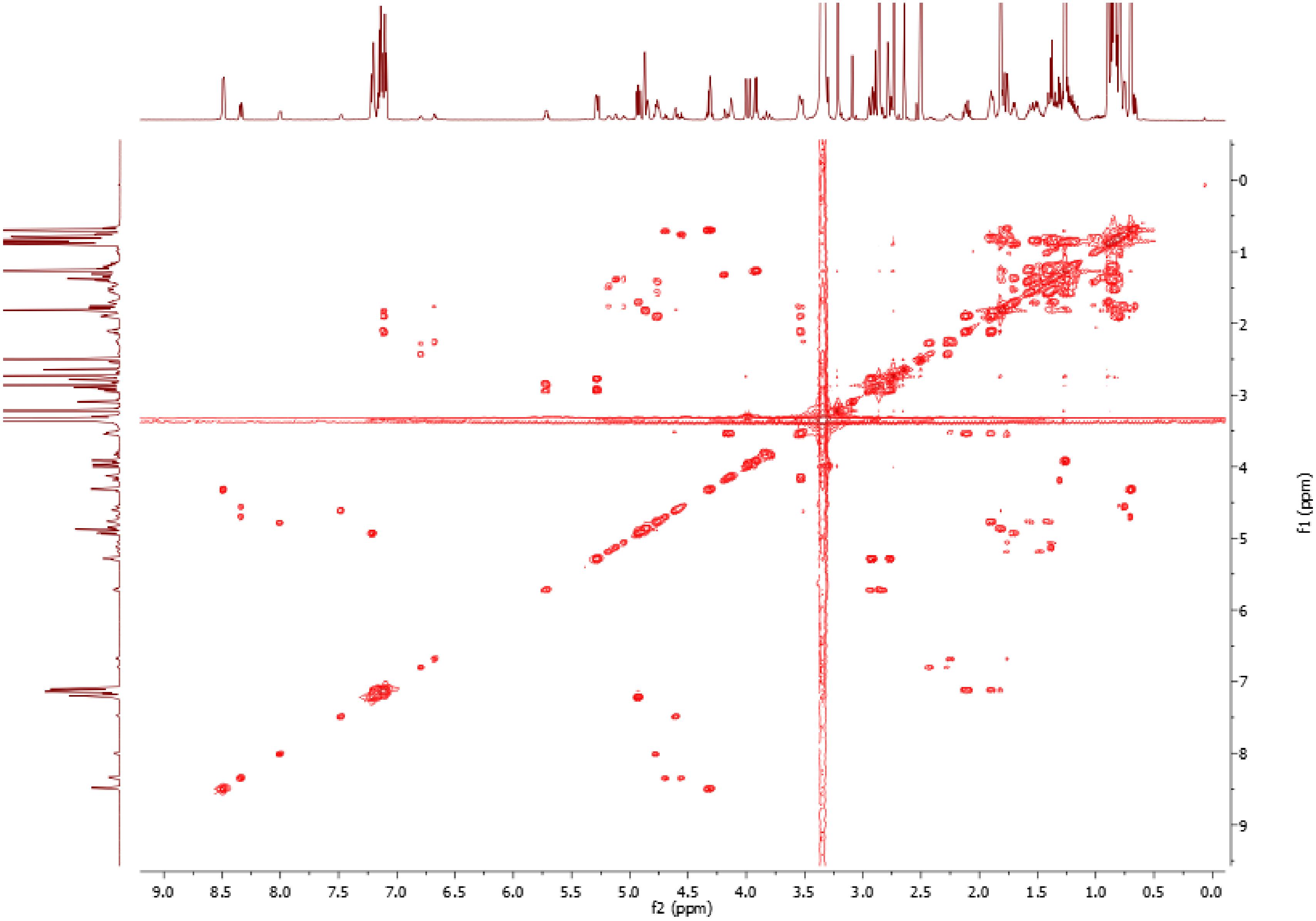


COSY spectrum of lagunamide D in $(CD_3)_2SO$ (600 MHz) at 27 °C.

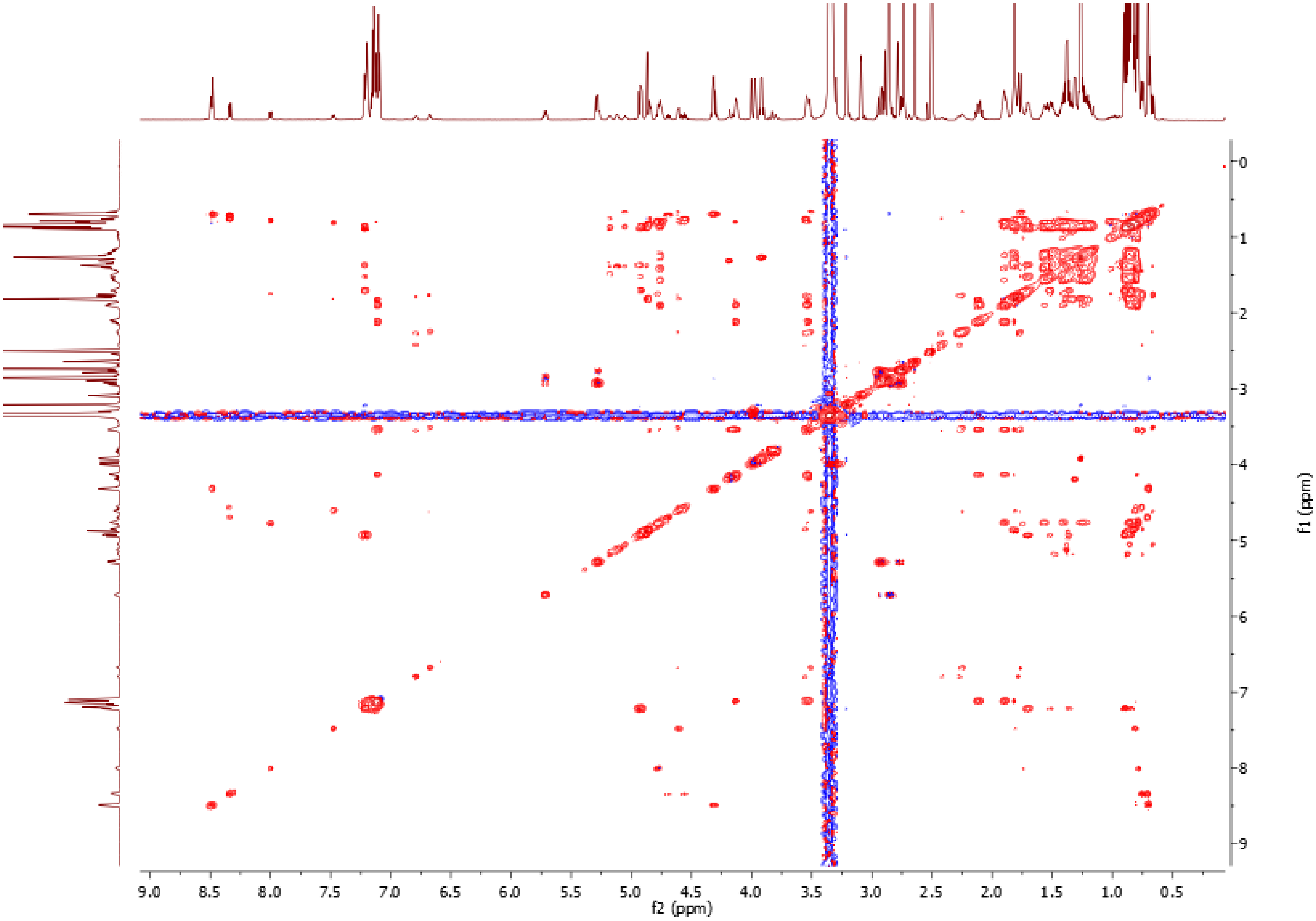


TOCSY spectrum of lagunamide D in $(CD_3)_2SO$ (600 MHz) at 27 °C.

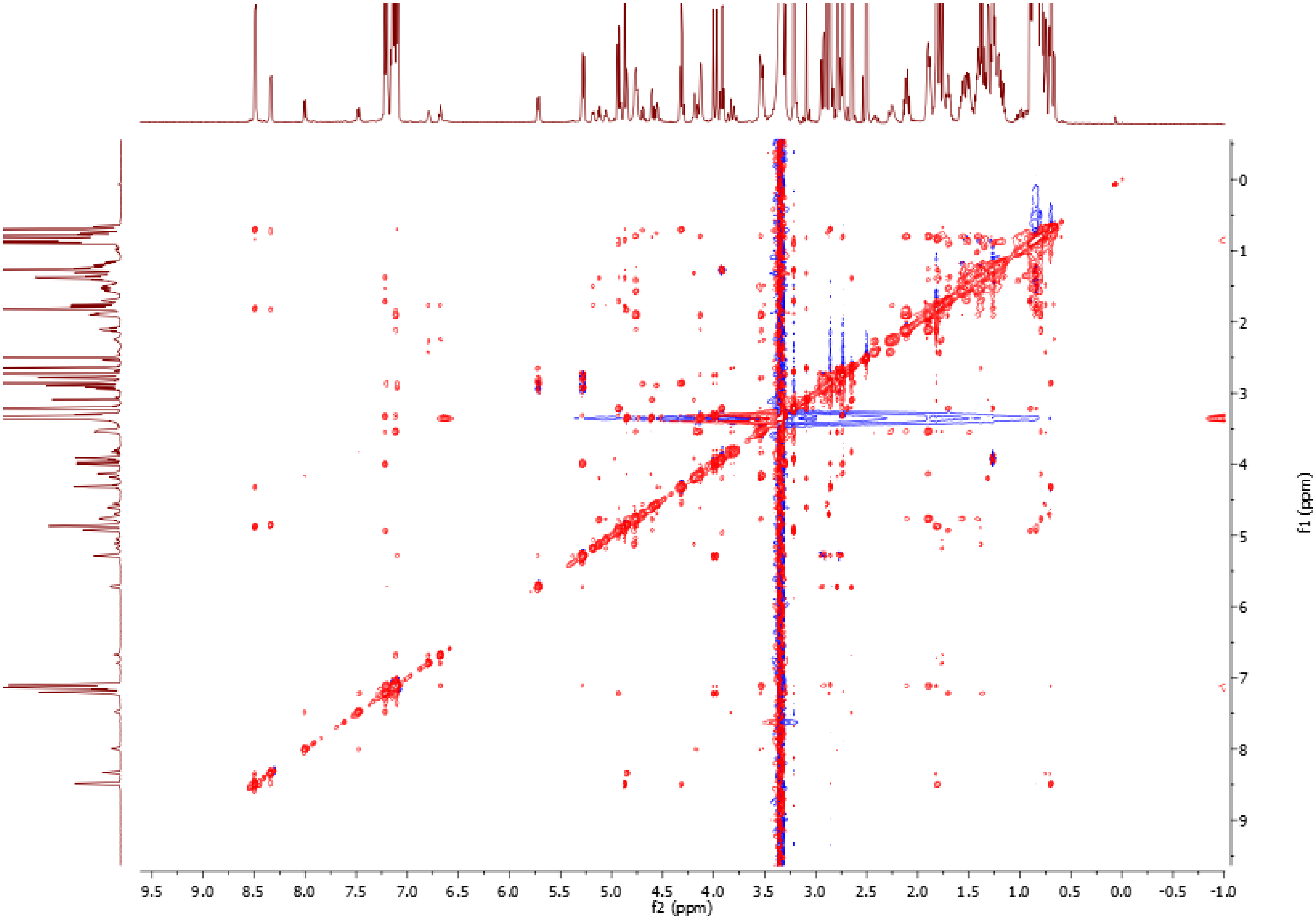


NOESY spectrum of lagunamide D in $(CD_3)_2SO$ (600 MHz) at 27 °C.

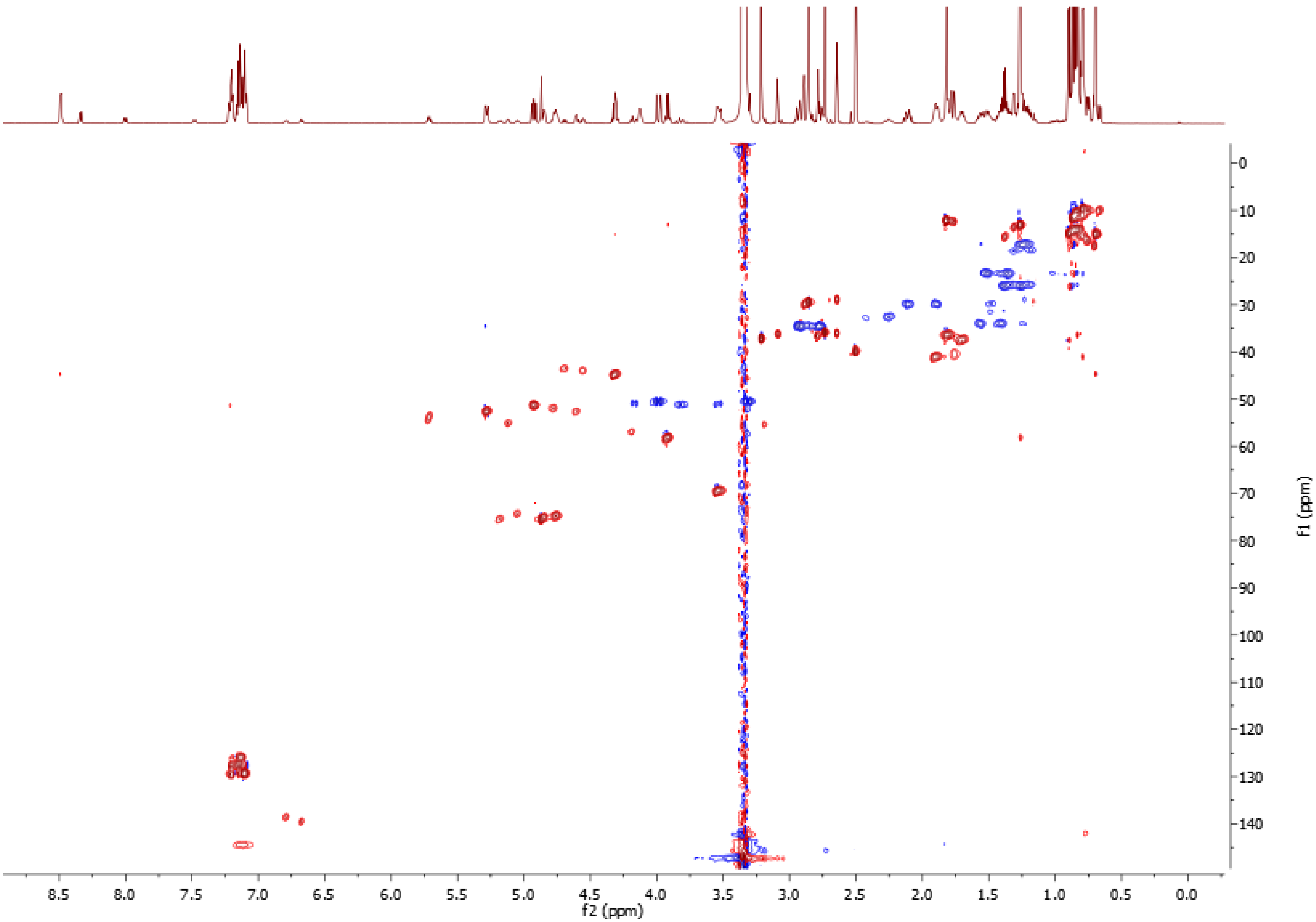


HSQC spectrum of lagunamide D in $(CD_3)_2SO$ (600 MHz) at 27 °C.

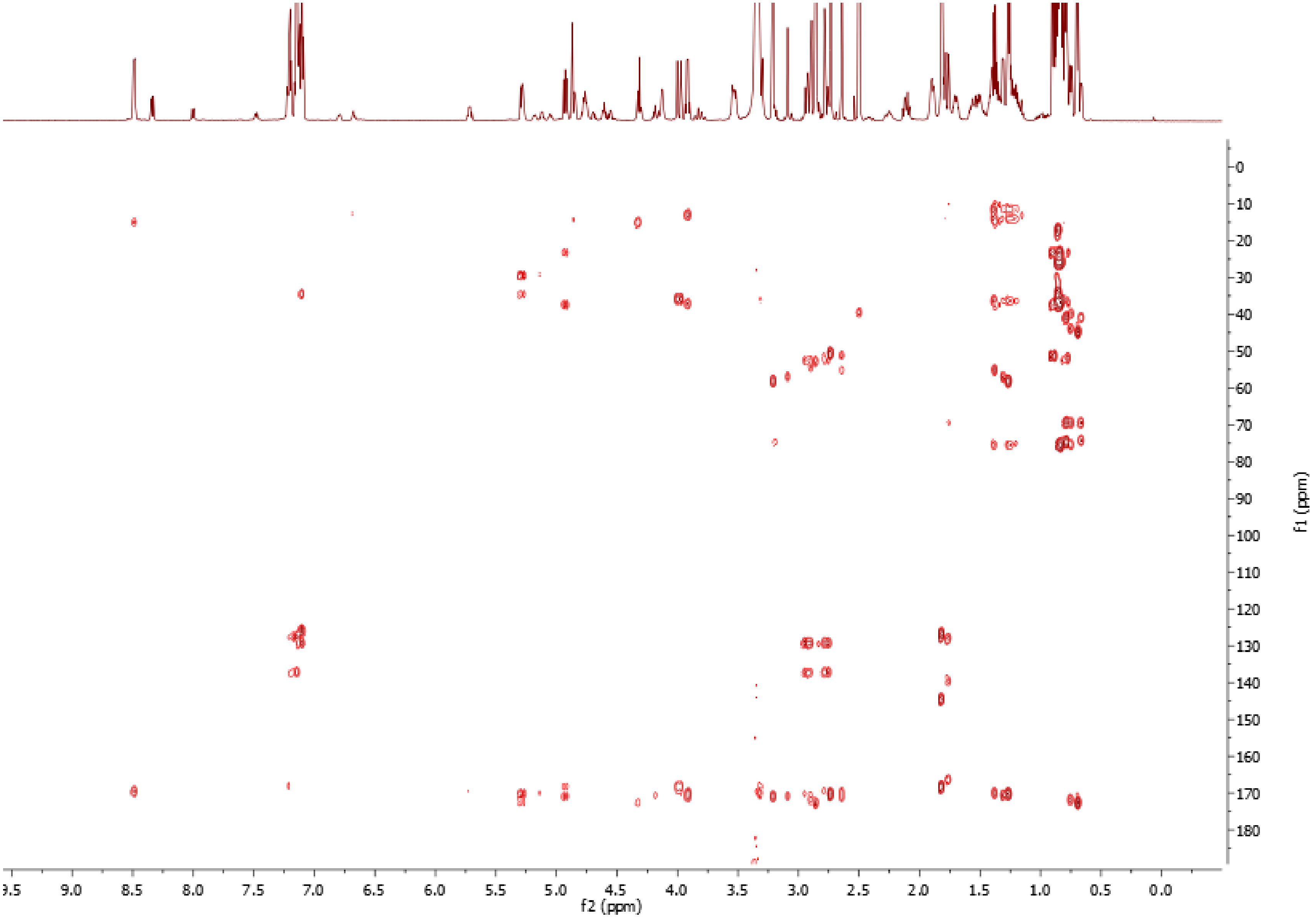


HMBC spectrum (optimized for $^{n}J$ = 7 Hz) of lagunamide D in $(CD_3)_2SO$ (600 MHz) at 27 °C.

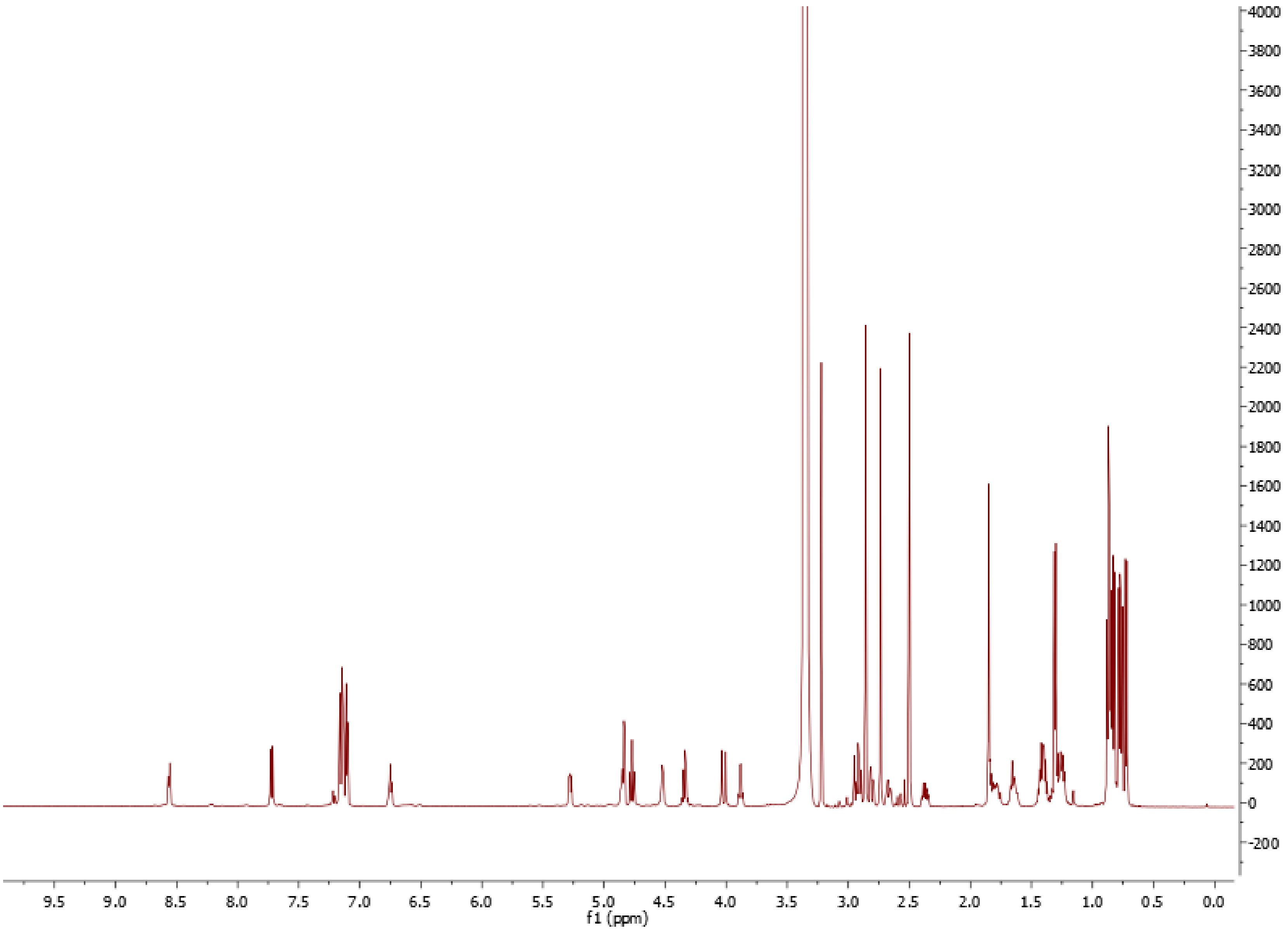


$^{1}$H NMR spectrum of lagunamide D’ in $(CD_3)_2SO$ (600 MHz) at 27 °C.

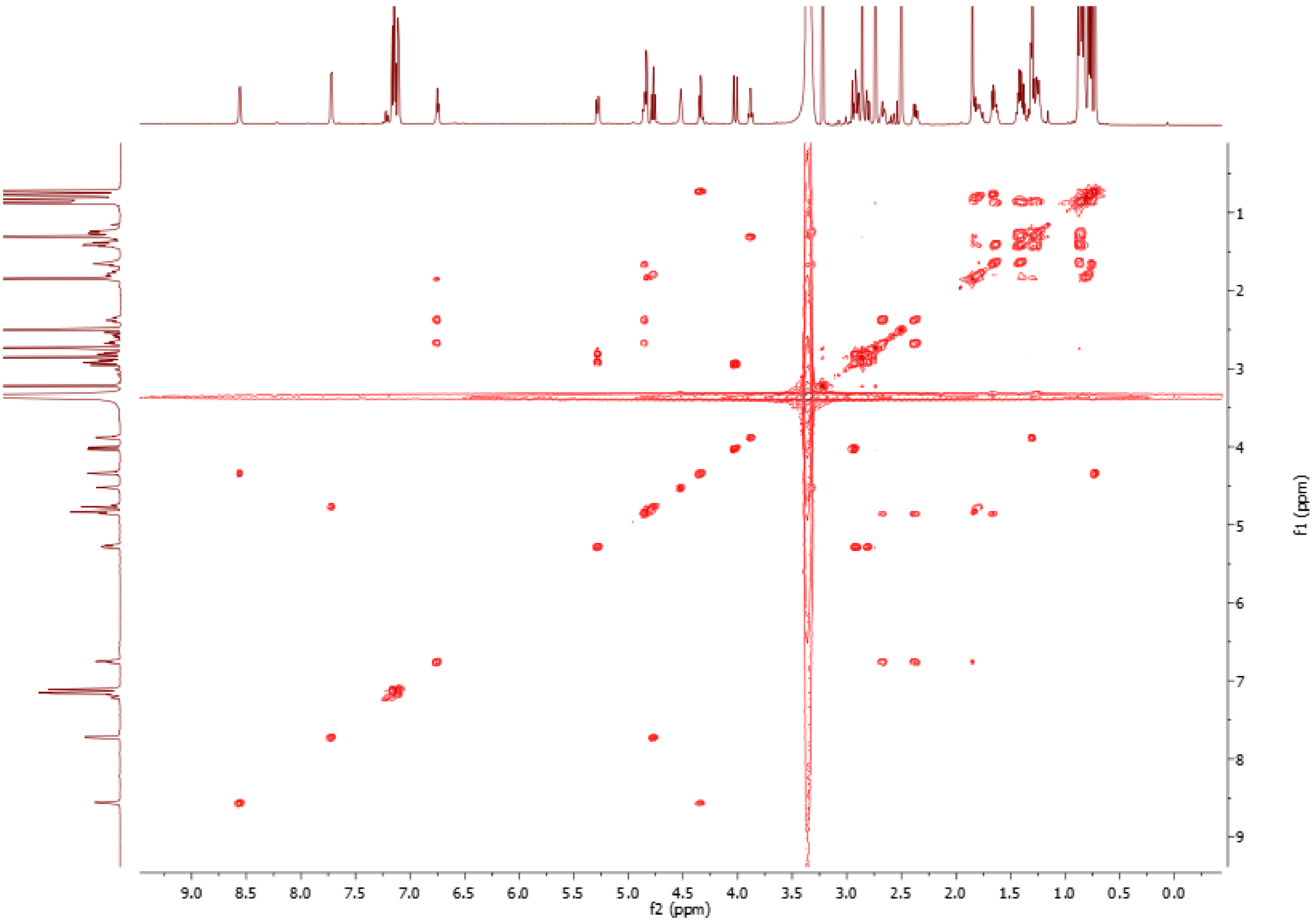


COSY spectrum of lagunamide D’ in $(CD_3)_2SO$ (600 MHz) at 27 °C.

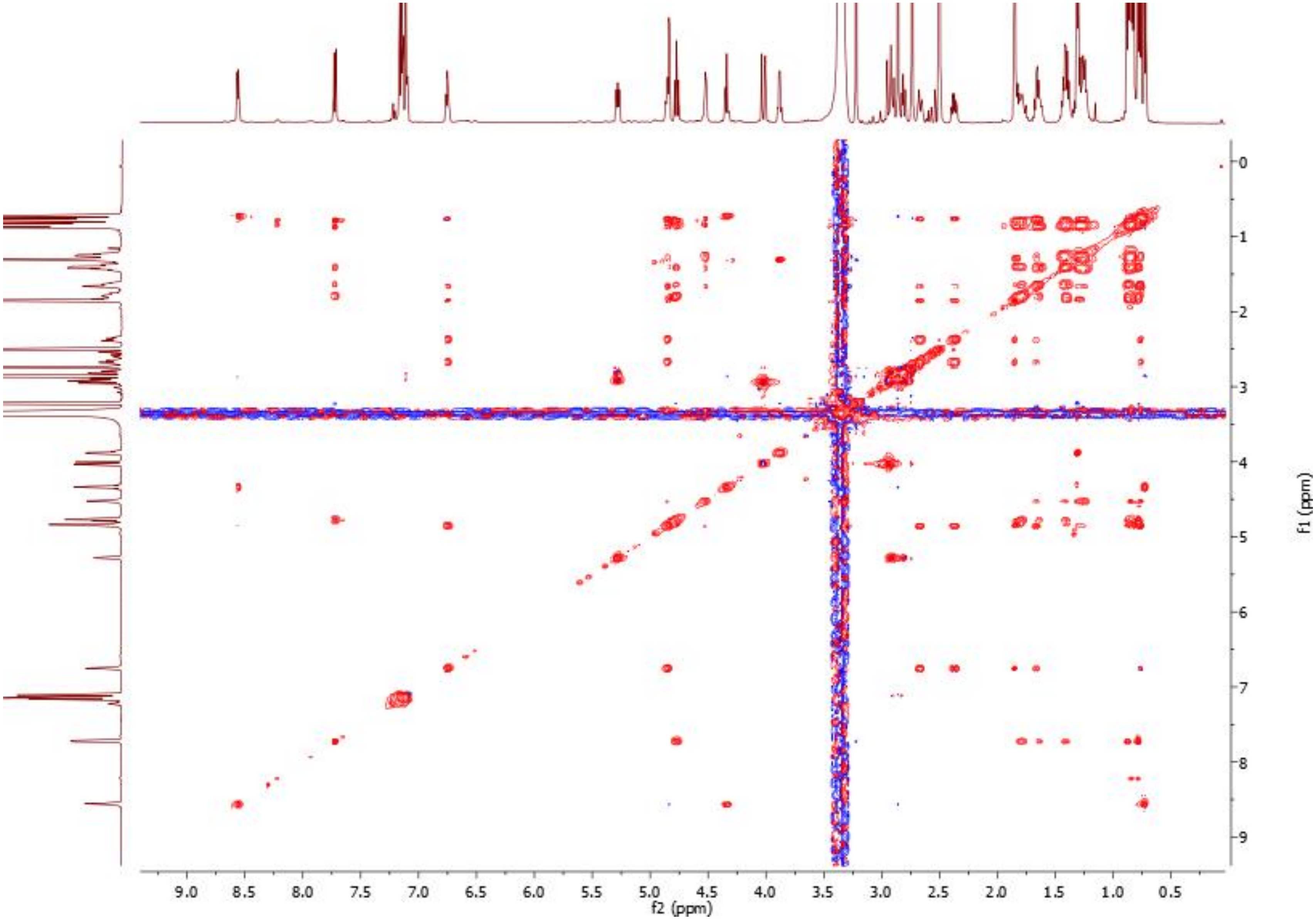


TOCSY spectrum of lagunamide D’ in $(CD_3)_2SO$ (600 MHz) at 27 °C.

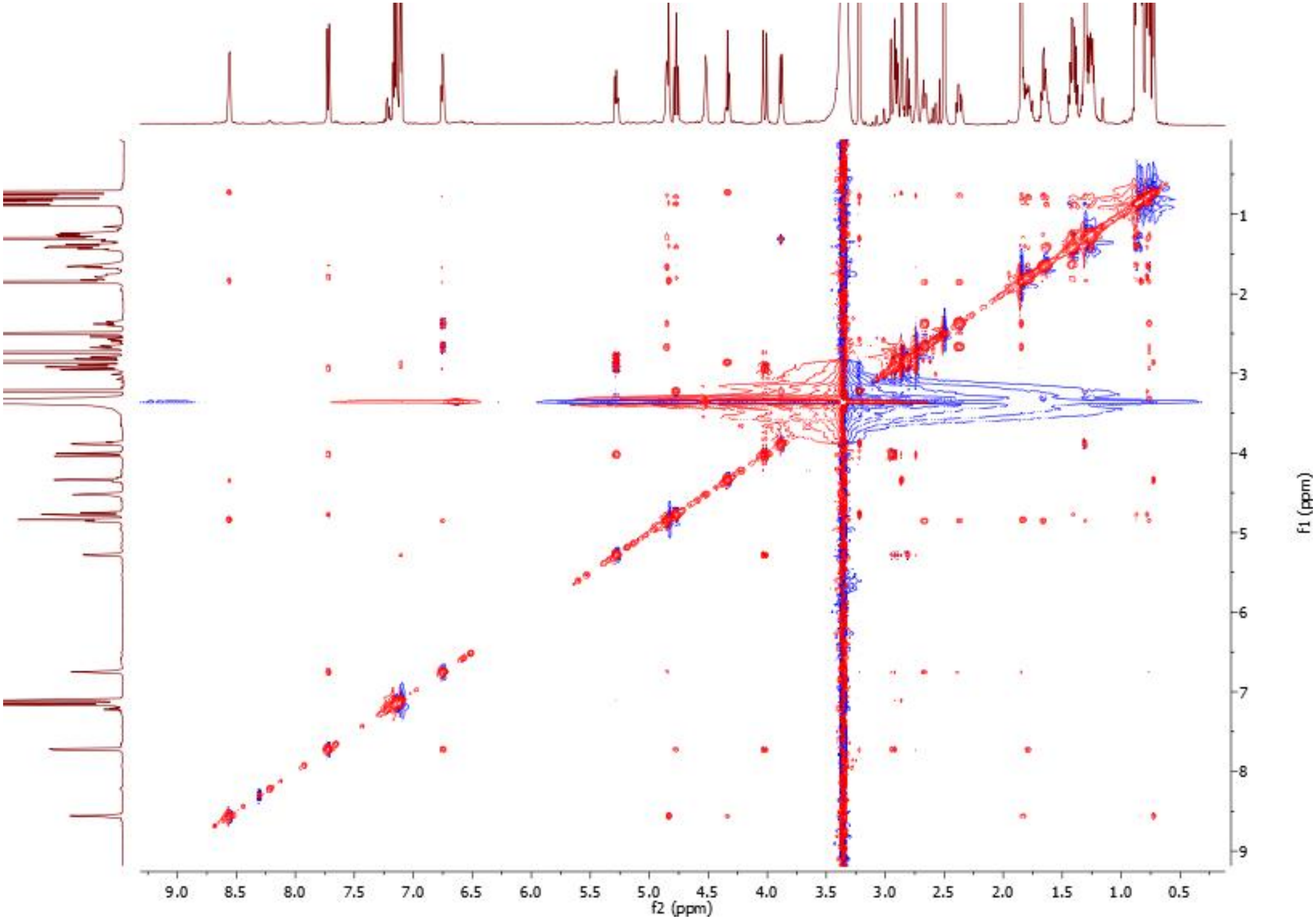


NOESY spectrum of lagunamide D’ in $(CD_3)_2SO$ (600 MHz) at 27 °C.

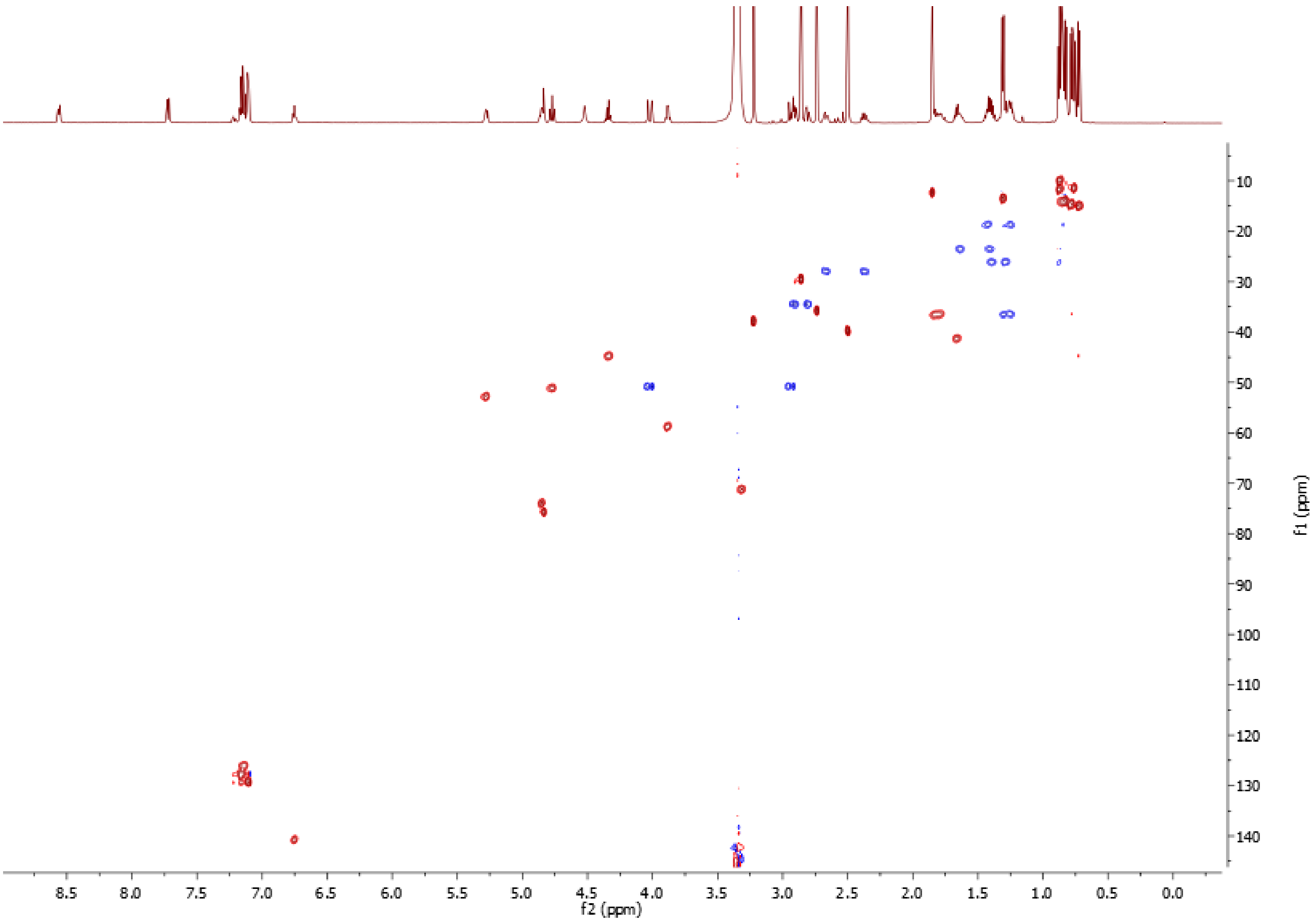


HSQC spectrum of lagunamide D’ in $(CD_3)_2SO$ (600 MHz) at 27 °C.

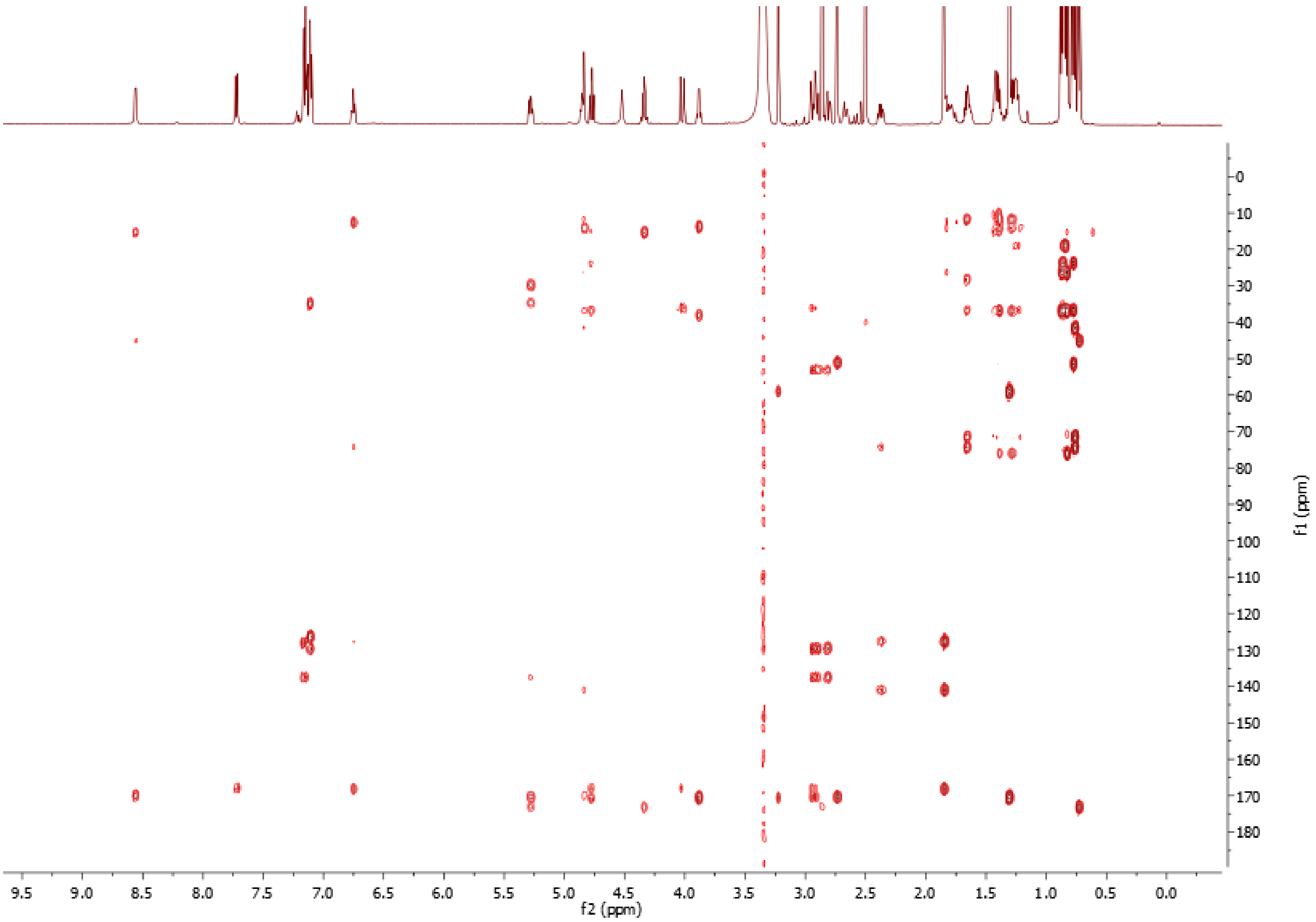


HMBC (optimized for $^{n}J$ = 7 Hz) spectrum of lagunamide D' in $(CD_3)_2SO$ (600 MHz) at 27 °C.

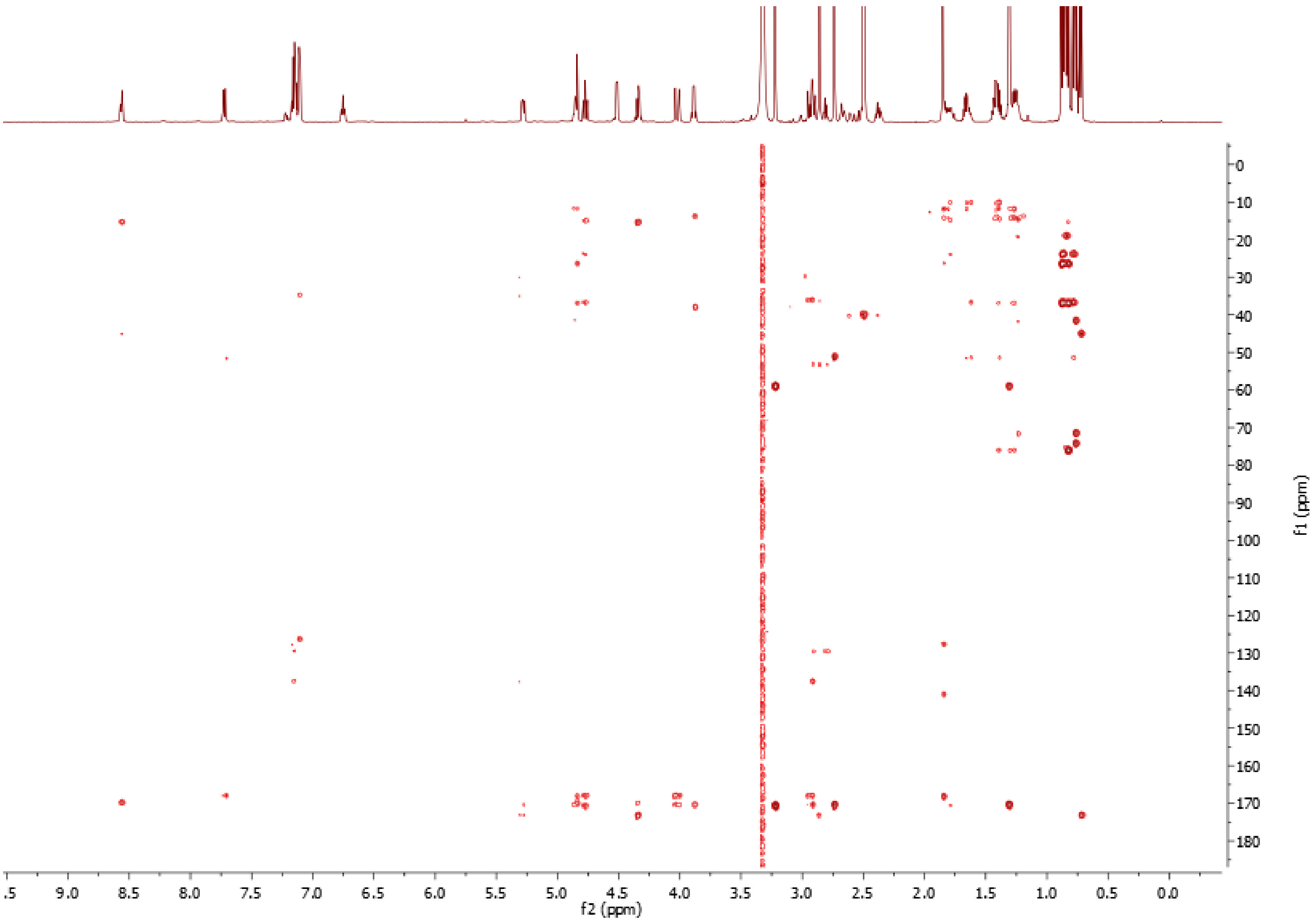


HMBC (optimized for $^{n}J$ = 3 Hz) spectrum of lagunamide D’ in $(CD_3)_2SO$ (600 MHz) at 27 °C.

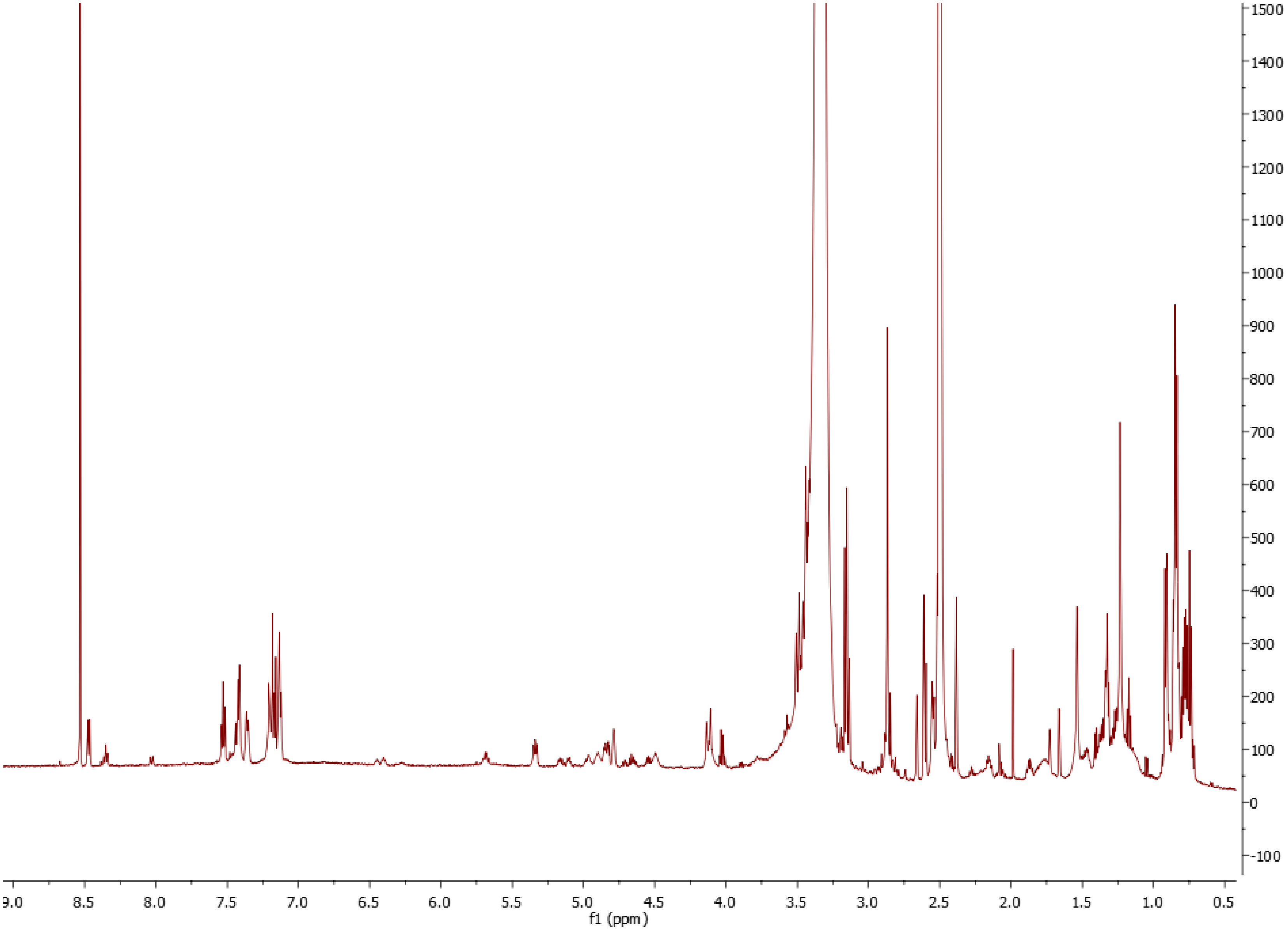


$^{1}$H NMR spectrum of the (*S*)-Mosher ester of lagunamide D in $(CD_3)_2SO$ (600 MHz) at 27 °C.

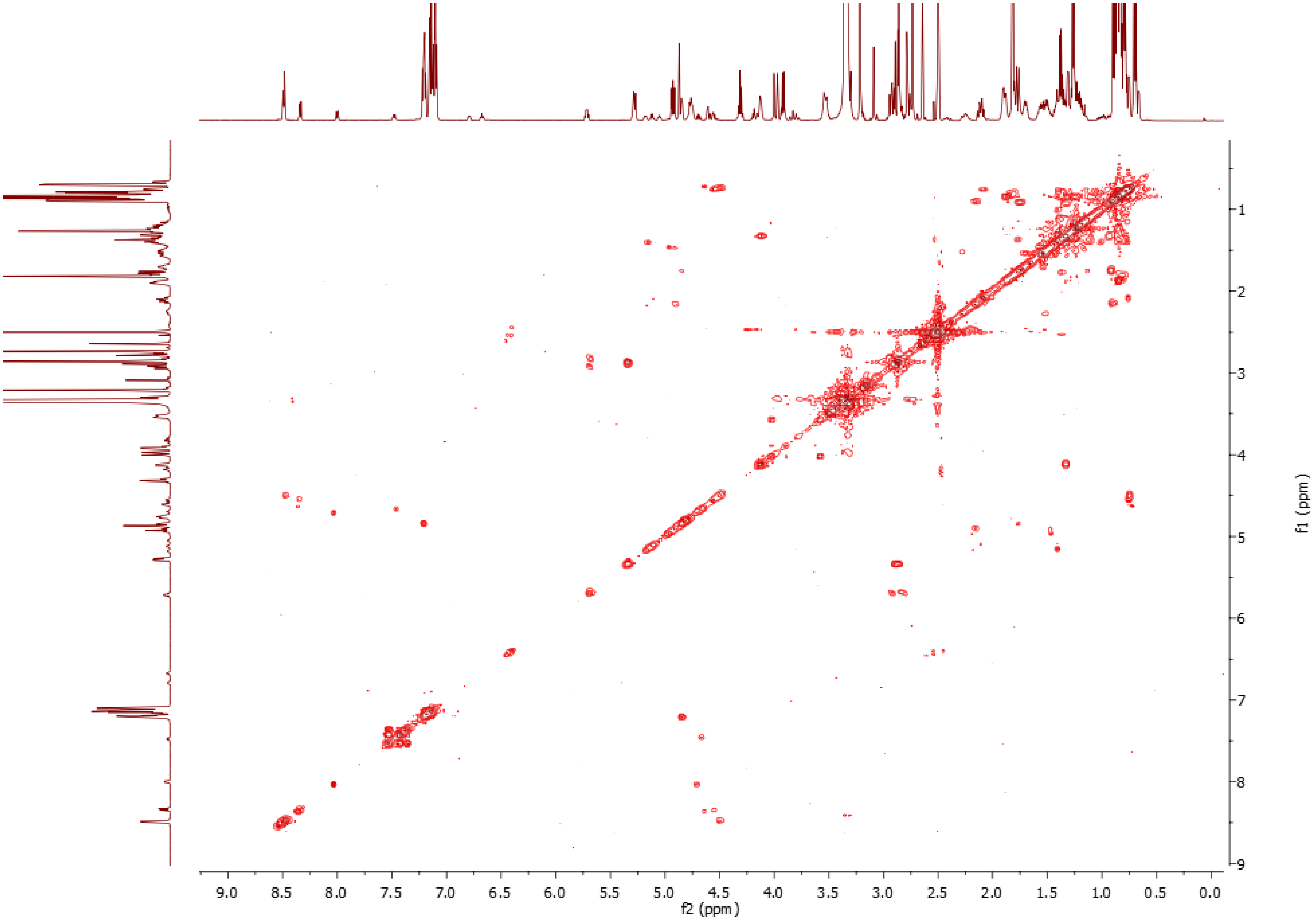


COSY spectrum of the (*S*)-Mosher ester of lagunamide D in $(CD_3)_2SO$ (600 MHz) at 27 °C.

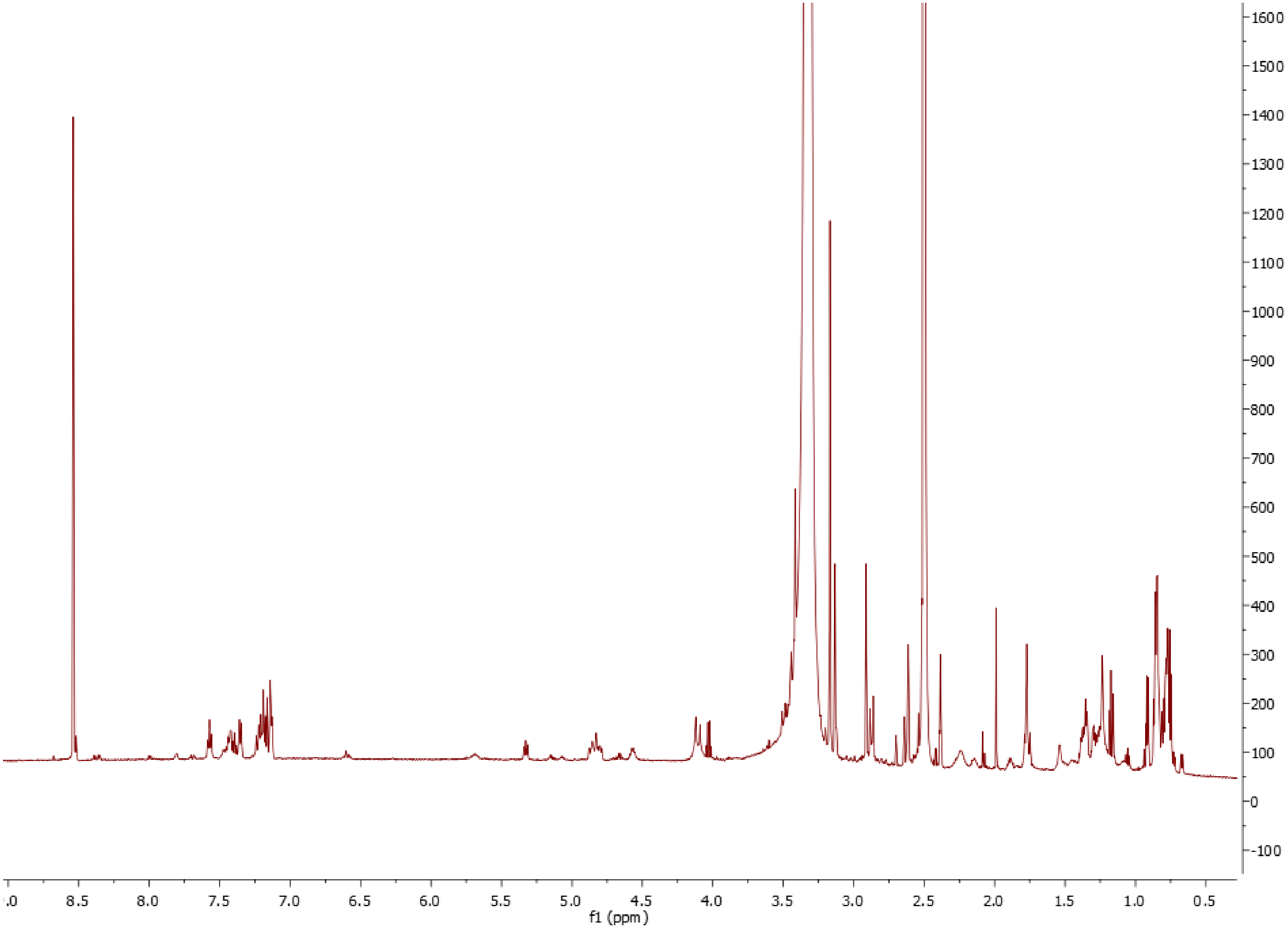


$^{1}$H NMR spectrum of the (*R*)-Mosher ester of lagunamide D in $(CD_3)_2SO$ (600 MHz) at 27 °C.

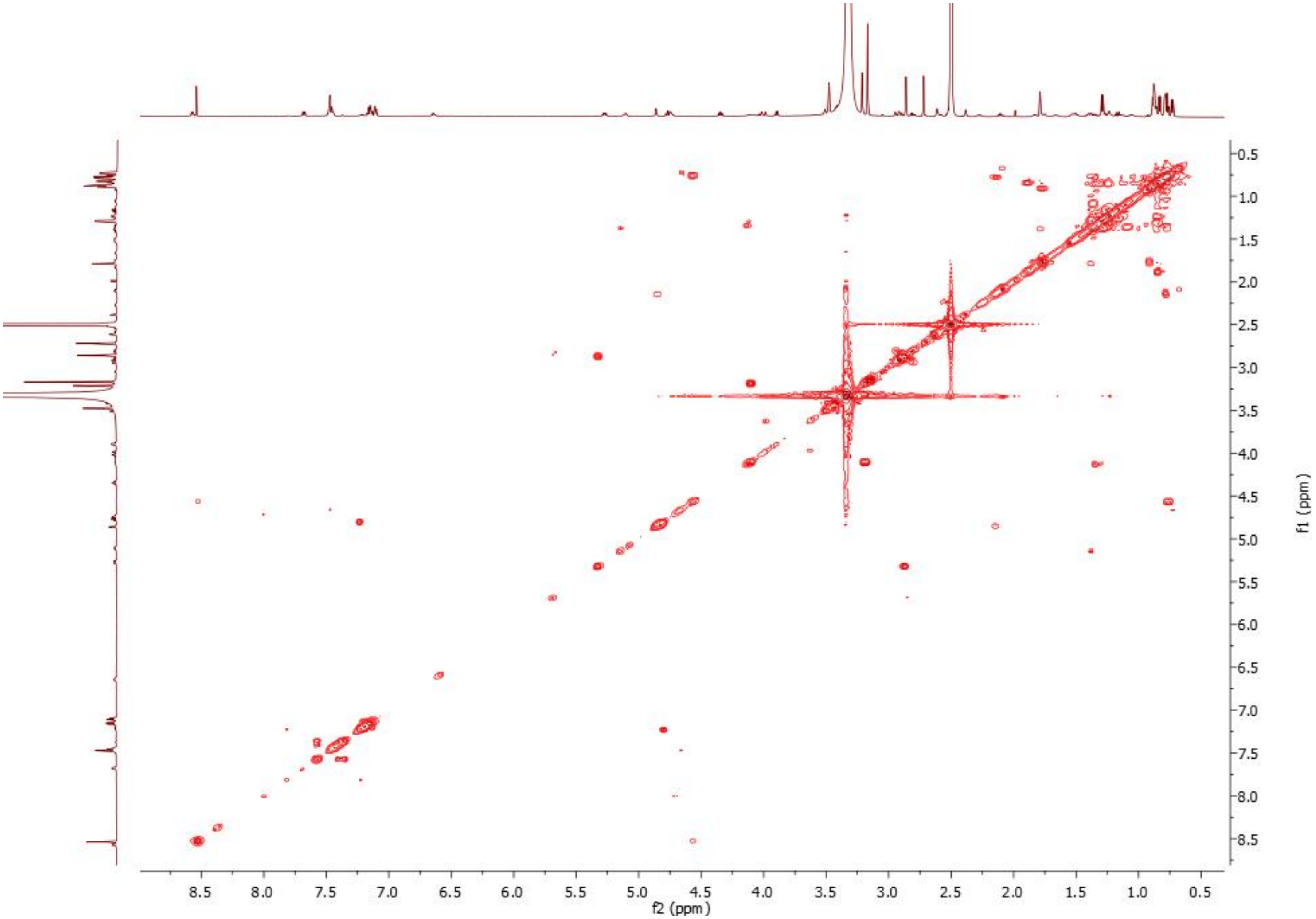


COSY spectrum of the (*R*)-Mosher ester of lagunamide D in $(CD_3)_2SO$ (600 MHz) at 27 °C.

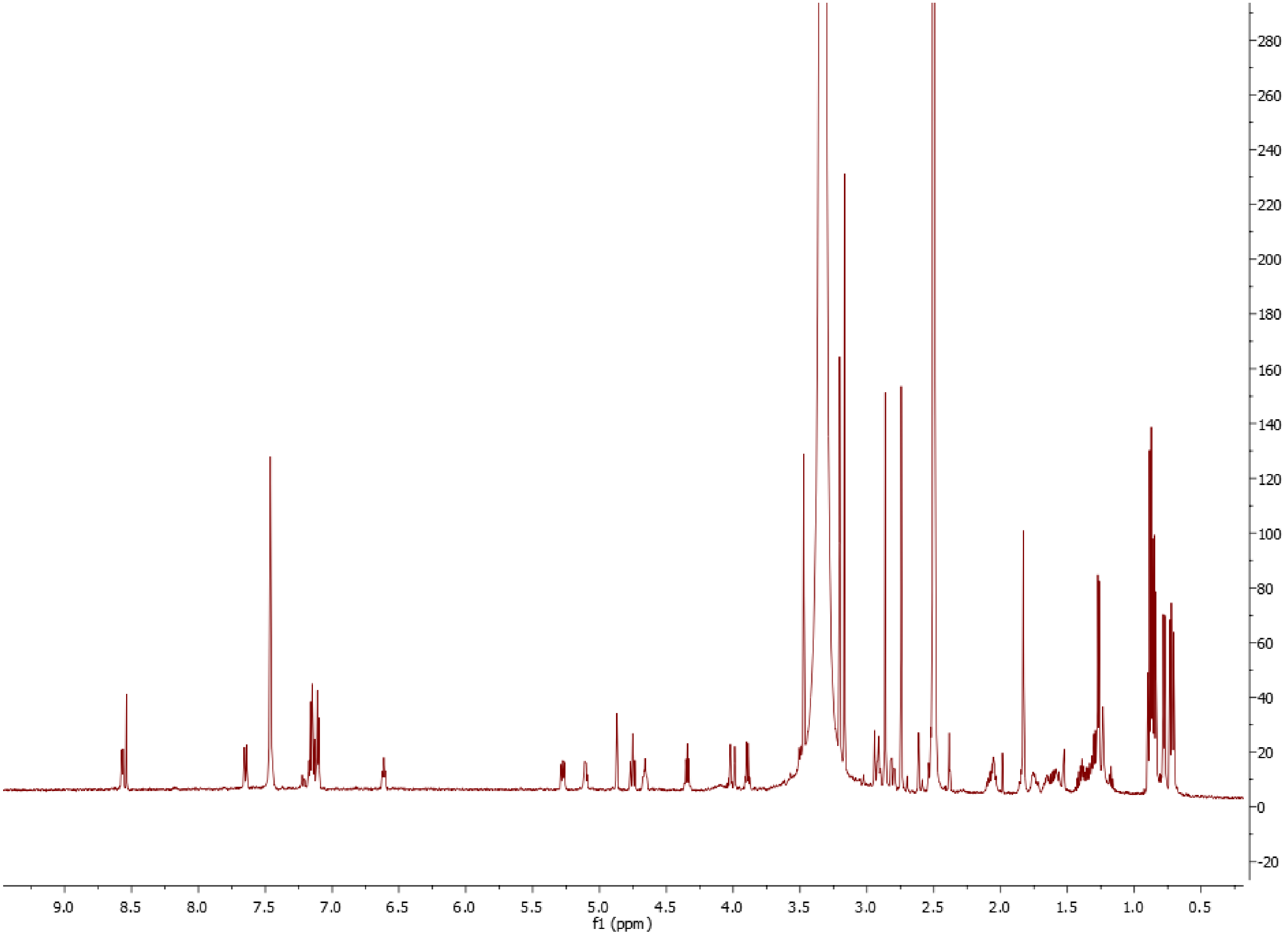


$^1$H NMR spectrum of the (*S*)-Mosher ester of lagunamide D' in $(CD_3)_2SO$ (600 MHz) at 27 °C.

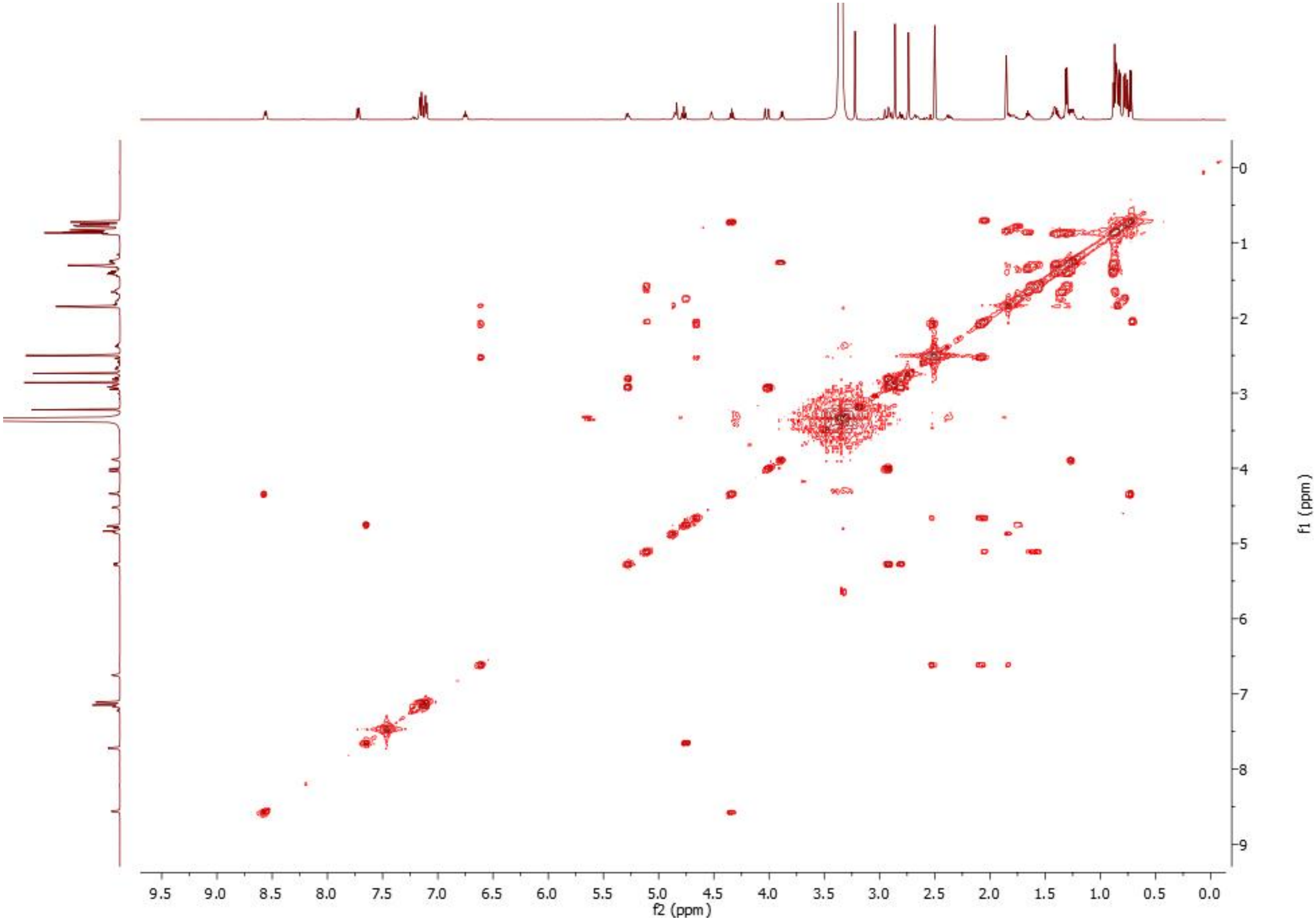


COSY spectrum of the (*S*)-Mosher ester of lagunamide D’ in $(CD_3)_2SO$ (600 MHz) at 27 °C.

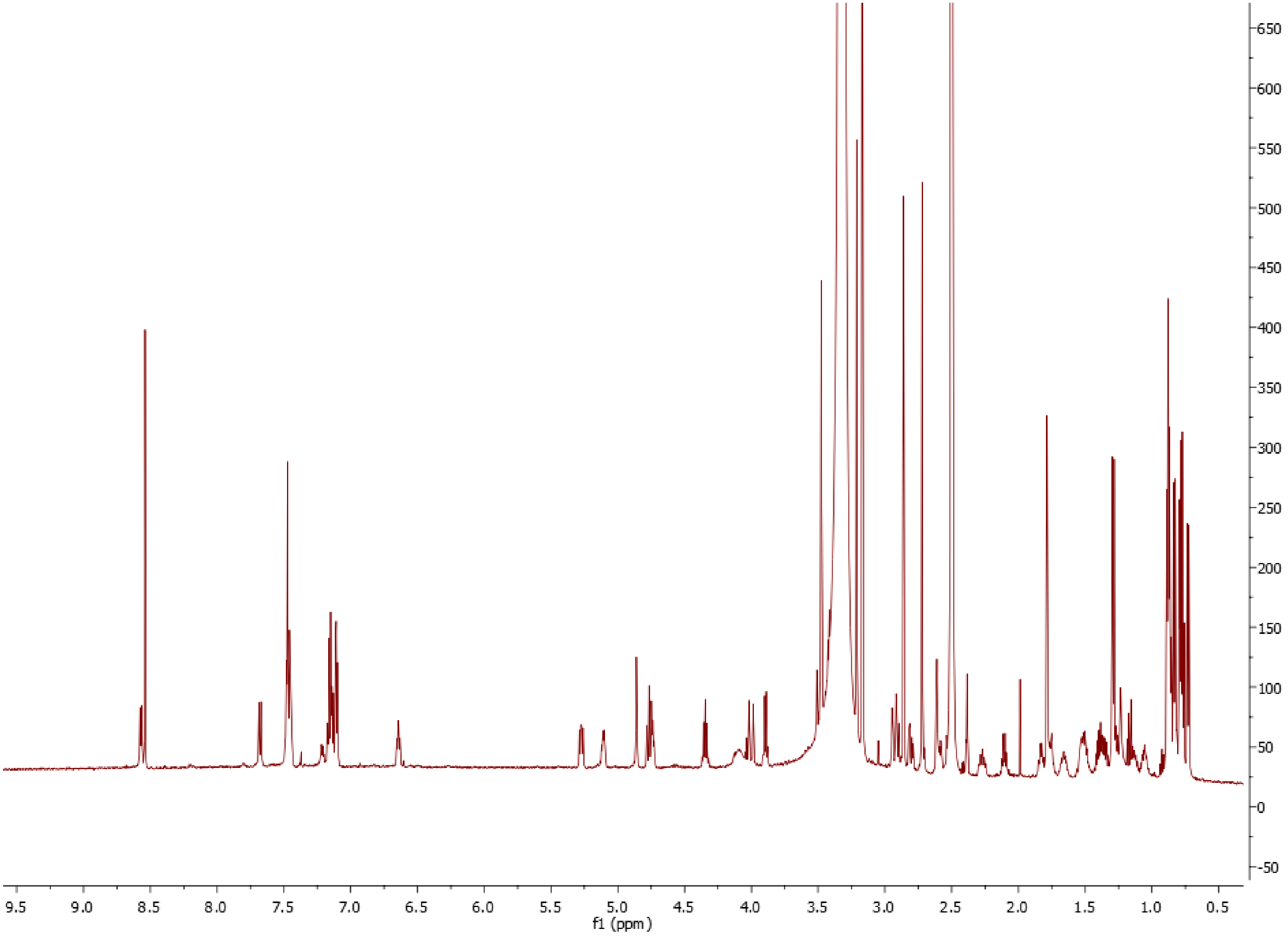


$^{1}$H NMR spectrum of the (*R*)-Mosher ester of lagunamide D’ in $(CD_3)_2SO$ (600 MHz) at 27 °C.

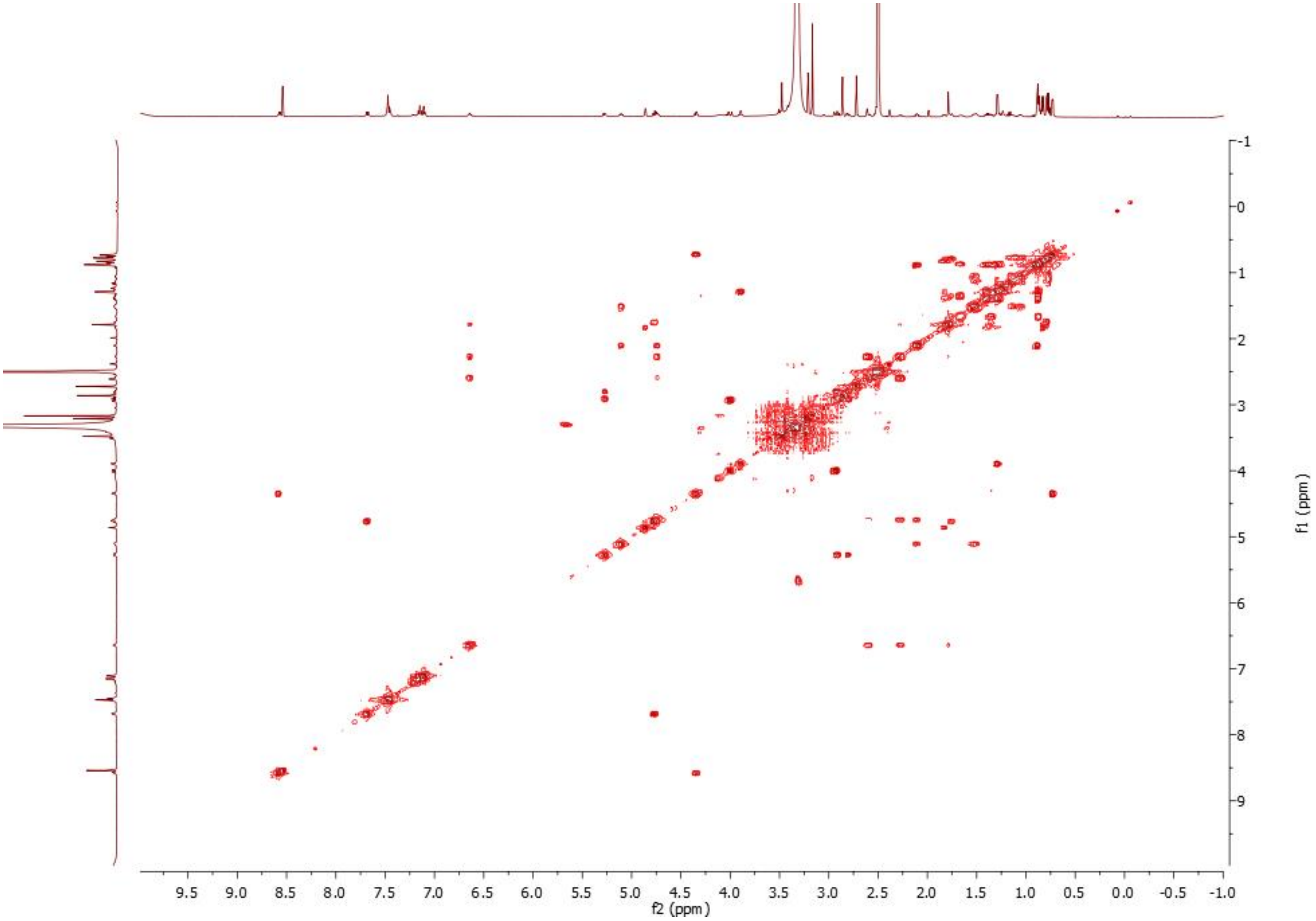


COSY spectrum of the (*R*)-Mosher ester of lagunamide D’ in $(CD_3)_2SO$ (600 MHz) at 27 °C.
